# MED19 encodes two unique protein isoforms that confer prostate cancer growth under low androgen through distinct gene expression programs

**DOI:** 10.1038/s41598-023-45199-9

**Published:** 2023-10-25

**Authors:** Rachel Ruoff, Hannah Weber, Ying Wang, Hongying Huang, Ellen Shapiro, David Fenyö, Michael J. Garabedian

**Affiliations:** 1 Department of Microbiology, New York University Grossman School of Medicine, New York, NY 10016 USA; 2 Institute for Systems Genetics, New York University Grossman School of Medicine, New York, NY 10016 USA; 3 Department of Biochemistry and Molecular Pharmacology, New York University Grossman School of Medicine, New York, NY 10016 USA; 4 Department of Urology, New York University Grossman School of Medicine, New York, NY 10016 USA

**Keywords:** Prostate cancer, Transcriptomics, Cancer models

## Abstract

MED19, a component of the mediator complex and a co-regulator of the androgen receptor (AR), is pivotal in prostate cancer cell proliferation. MED19 has two isoforms: a full-length “canonical” and a shorter “alternative” variant. Specific antibodies were developed to investigate these isoforms. Both exhibit similar expression in normal prostate development and adult prostate tissue, but the canonical isoform is elevated in prostate adenocarcinomas. Overexpression of canonical MED19 in LNCaP cells promotes growth under conditions of androgen deprivation in vitro and in vivo, mirroring earlier findings with alternative MED19-overexpressing LNCaP cells. Interestingly, alternative MED19 cells displayed strong colony formation in clonogenic assays under conditions of androgen deprivation, while canonical MED19 cells did not, suggesting distinct functional roles. These isoforms also modulated gene expression differently. Canonical MED19 triggered genes related to extracellular matrix remodeling while suppressing those involved in androgen-inactivating glucuronidation. In contrast, alternative MED19 elevated genes tied to cell movement and reduced those associated with cell adhesion and differentiation. The ratio of MED19 isoform expression in prostate cancers shifts with the disease stage. Early-stage cancers exhibit higher canonical MED19 expression than alternative MED19, consistent with canonical MED19’s ability to promote cell proliferation under androgen deprivation. Conversely, alternative MED19 levels were higher in later-stage metastatic prostate cancer than in canonical MED19, reflecting alternative MED19’s capability to enhance cell migration and autonomous cell growth. Our findings suggest that MED19 isoforms play unique roles in prostate cancer progression and highlights MED19 as a potential therapeutic target for both early and late-stage prostate cancer.

## Introduction

The Mediator complex conveys functional information from enhancer-bound transcription factors to the basal transcription machinery at the promoter^1–3^ and supports transcriptional elongation^4^ and RNA polymerase II (RNAP II) recycling^5^. The Mediator complex consists of 33 subunits in mammals. The complex’s composition can vary, with distinct subunits interacting with various transcription factors in a cell-specific manner^6,7^. Our group and others have established the significance of MED19 in prostate cancer cell proliferation^8,9^. We previously found that reducing MED19 levels inhibits androgen receptor (AR) transcriptional activity and the proliferation of androgen-dependent LNCaP cells and androgen-independent LNCaP-abl cells^8^. MED19 mRNA exhibits increased expression in primary and metastatic prostate cancer, correlating with a lower overall survival^8,9^.

Cryo-EM of the Mediator complex revealed a tripartite structure composed of head, middle, and tail modules. MED19 is located in the “hook” section of the middle module, where it interacts with MED14, MED10, and the N-terminal region of MED1^10–13^. This middle hook domain provides a surface that accommodates and orients the TFIIH complex, which contains CDK7, to phosphorylate the carboxy-terminal domain (CTD) of RNAP II^14^, with several MED19 N-terminal residues crosslinking to the CTD^1,15^. While depletion of MED19 reduced cell viability in AR-dependent prostate cancer cells^8^, this was not the case in mouse T cells, B cells, and embryonic stem cells, where deletion of MED19 was not essential for viability^16^. These findings suggests that MED19 is not a universally required subunit for cell viability like the “backbone” subunit MED14^16^. Instead, the removal of MED19 in B cells resulted in the up-and down-regulation of approximately 2000 genes compared to control cells^16^. Therefore, MED19 is crucial in controlling specific gene subsets and directs prostate cancer survival and proliferation by regulating AR transcriptional activity.

MED19 has two predicted isoforms: a full-length canonical version (244 amino acids) and a shorter variant we refer to as “alternative” MED19 (194 amino acids), formed by alternate splice site selection resulting in the loss of the last 50 amino acids in the canonical MED19 and the addition of a unique sequence of four amino acids. Both MED19 isoforms at the mRNA level are found in circulating CD133+ endothelial progenitor cells, with the alternative MED19 showing reduced expression in differentiated endothelial cells^17^. Considering that alternative MED19 has high expression in endothelial progenitor cells that decreases upon differentiation and that MED19 protein levels are elevated in prostate tumors and associated with high grade and poor differentiation in other cancers^18,19^, we hypothesized that alternative MED19 might play a role in prostate cancer progression. Indeed, we previously reported that overexpression of alternative MED19 in androgen-dependent prostate cancer cells caused early-stage androgen-dependent prostate cancer cells to become androgen-independent by modifying AR-dependent gene expression^19^. However, the roles of canonical MED19 in prostate cancer cell proliferation and gene regulation remain undetermined.

This study investigated the effects of canonical MED19 overexpression on prostate cancer cell proliferation, tumor growth, and gene expression. We compared these effects with those of alternative MED19. Additionally, we employed MED19 isoform-specific antibodies developed in our laboratory to explore the expression patterns of canonical MED19 and alternative MED19 proteins in prostate development, normal adult prostate tissue, and prostate cancer. Our results indicate that increased levels of canonical MED19 led to enhanced prostate cancer cell proliferation and tumor growth in low androgen conditions, resembling the impact observed with alternative MED19. However, canonical and alternative MED19 isoforms govern distinct sets of genes, and alternative MED19, but not canonical MED19, can promote cell migration and autonomous cell growth. Consequently, the upregulation of MED19 isoforms confers phenotypes associated with prostate cancer progression by altering gene expression. This study marks the first characterization of MED19 isoforms in cancer, shedding light on their distinct functions in prostate cancer.

## Results

### MED19 produces distinct protein isoforms found in prostate tissue and prostate cancer cells

**Figure 1:**
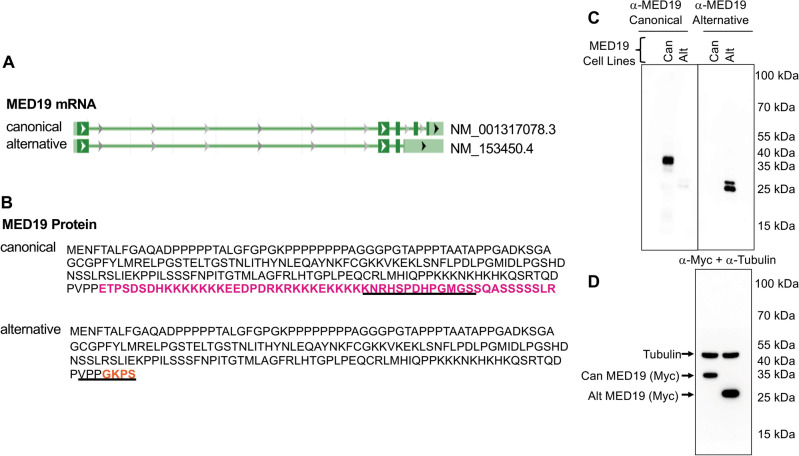
Expression of two mRNA isoforms from the MED19 locus that encode distinct proteins. (**A**) Analysis of the human genome predicts two mRNA variants for MED19 that have the potential to code for proteins. (**B**) The canonical MED19 isoform has five exons encoding a 244 amino acid protein with a sequence of 50 amino acids at its C-terminus found exclusively in this isoform (highlighted in pink). A spliced alternative MED19 mRNA lacking exons 4–5 encoding a 194 amino acid protein that contains four unique amino acids at its C-terminus due to alternative splice site selection from exon 3 (highlighted in orange) and includes a longer 3′ UTR. Affinity-purified rabbit polyclonal antibodies were generated against canonical MED19 corresponding to residues 222–234 found exclusively in this isoform and alternative MED19 corresponding to residues 186–194, including the four unique amino acids present in alternative MED19 (underlined in panel **B**). (**C**) Western blot of LNCaP cells expressing canonical MED19 or alternative MED19 using isoform-specific antibodies. The same amount of whole cell lysates from LNCaP cells expressing canonical and alternative MED19 were run in duplicate on the same gel, transferred, cut in two, and blotted separately with affinity purified antibody against canonical MED19 (top left panel) or alternative MED19 (top right panel). The images of the western blots shown for MED19 isoforms were exposed for the same length of time (0.5 s). (**D**) Filter from Panel (**C**) (right panel) was probed with an antibody to the MYC tag common to both MED19 isoforms and tubulin, which serves as a loading control (exposure time = 0.3 s). Chemiluminescence was captured via the iBright Imaging System using the Auto-Exposure option. Shown are the raw, unprocessed images. The imaging parameters are as follows: Zoom level = 1.2×; Focus level = 246; Resolution = 5 × 5; Exposure mode = normal; Exposure times: 0.3–0.5 s. Longer exposures are shown in Fig. S11A.

Analysis of the human genome indicates two MED19 variants with protein-coding potential (Fig. 1A). The canonical MED19 isoform (Accession NM_001317078.4) consists of five exons and produces a 244-amino acid protein with a predicted molecular weight of 26.3 kD. An alternatively spliced MED19 variant (Accession NM_153450.4) omits exons 4–5 and possesses an extended 3ʹ UTR, leading to a 194-amino acid protein with an estimated molecular weight of 20.4 kD (Fig. 1B). This MED19 isoform also includes four unique amino acids at its C-terminus, resulting from an alternate splice site choice in exon 3 (Fig. 1B). We introduced the term “alternative MED19” to refer to the shorter variant of MED19, thereby differentiating it from the standard, or canonical, MED19 isoform.

To determine whether MED19 isoforms are present at the protein level, we created isoform-specific antibodies to identify canonical MED19 or alternative MED19. We generated rabbit polyclonal antibodies against canonical MED19, which correspond to residues 222–234 exclusive to this isoform, and alternative MED19, which correspond to residues 186–194 containing the four unique amino acids found in this variant (Fig. 1B). Both antibodies were affinity purified using their respective peptides.

To assess the antibodies’ capability of detecting MED19 isoforms, we performed western blots on LNCaP cell extracts expressing MYC-tagged versions of either the canonical MED19 or alternative MED19. As shown in Fig. 1C, equal amounts of whole cell lysates from LNCaP cells, expressing either canonical or alternative MED19, were run in parallel on the same gel. These were then transferred to a membrane, which was cut into two sections. Each section was independently blotted with antibodies for canonical MED19 or alternative MED19. The individual blots were placed side by side and exposed for the same length of time. We found that the anti-canonical MED19 antibody recognized canonical MED19 and showed minimal reactivity towards the alternative MED19, whereas the antibody targeting the alternative MED19 recognized alternative MED19 and lacked reactivity towards the canonical MED19. Alternative MED19 appears as a doublet, which could reflect a post-translational modification. Immunoblotting of the MED19 isoforms using the MYC epitope common to each isoform revealed similar amounts of MED19 (Fig. 1C). Hence, these antibodies allow for the detection of MED19 protein isoforms and exhibit similar affinity towards their respective proteins.

**Figure 2:**
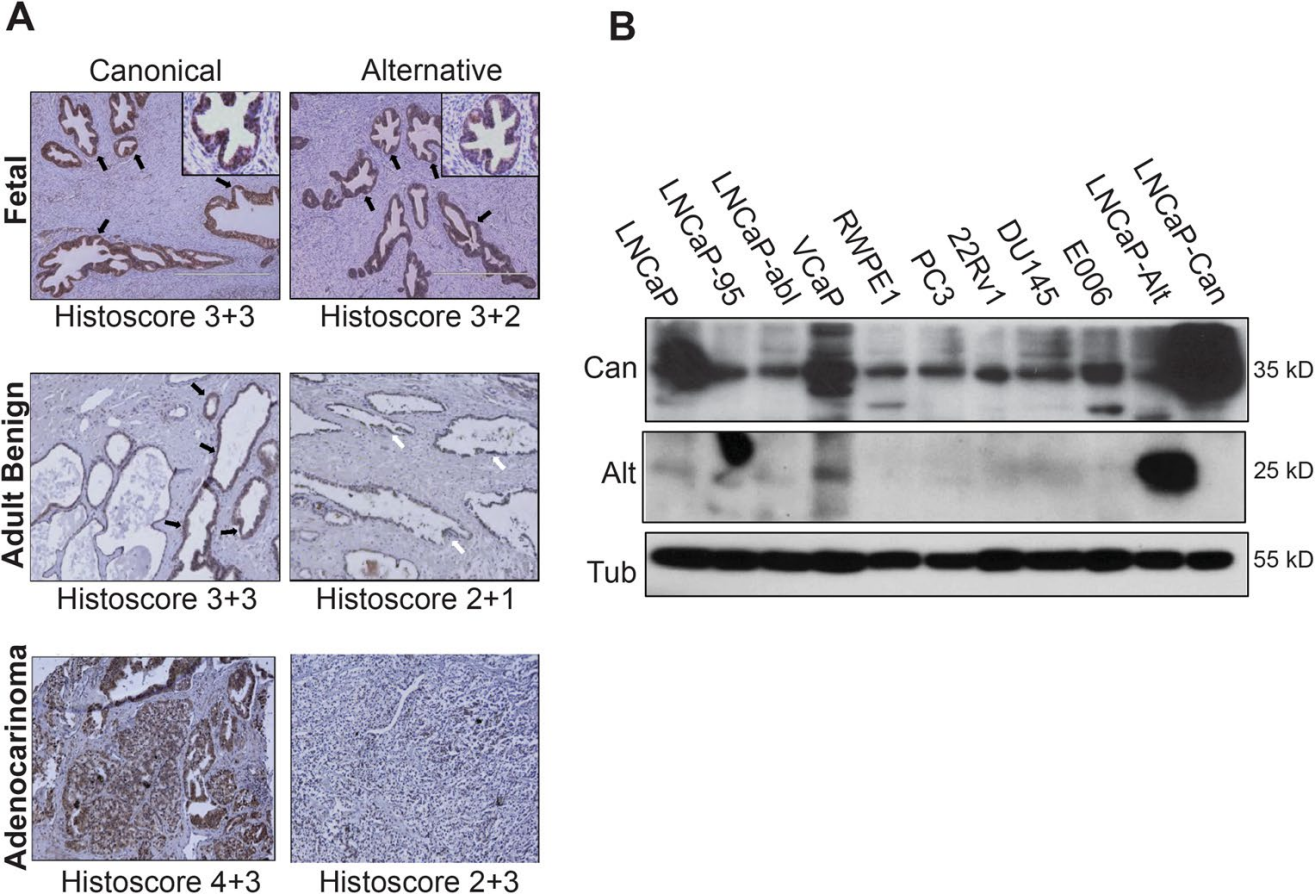
MED19 protein variants are present in prostate tissues and cells. (**A**) Expression of MED19 variants in normal and cancerous prostate tissues. Human fetal prostate tissue at 23 weeks (top panels, with IRB approval) was stained with affinity purified antibody against canonical MED19 or alternative MED19 (× 20 magnification; inset × 40 magnification). A commercially available prostate cancer tissue microarray (TMA) containing normal (n = 27) and adenocarcinoma samples (n = 18) was stained using antibodies specific for canonical and alternative MED19. Brown staining indicates MED19 presence (black arrows indicate strong staining, while white arrows indicate weaker staining). Hematoxylin (blue) was used as a counterstain. Histoscores are provided, with the first score representing the percentage of stained cells and the second score indicating staining intensity (1 = low; 4 = high). No staining was detected when using only the secondary antibody. (**B**) Western blot analysis of total protein lysates from the indicated prostate cancer cell lines using antibodies against canonical or alternative MED19 and tubulin. The uncropped blots are shown in Fig. S11B.

We next investigated the expression of MED19 protein isoforms in various stages of prostate development and cancer using immunohistochemistry (IHC) (Fig. 2A). Our results showed that both canonical and alternative MED19 were equally expressed in epithelial cells during early prostate development (histoscore 3 + 3 for canonical MED19 and 3 + 2 for alternative MED19) (Fig. 2A; top panels). In adult normal prostate tissue, we found that canonical MED19 was expressed at a higher level than alternative MED19 (histoscore 3 + 3 for canonical MED19 and 2 + 1 for alternative MED19) (Fig. 2A; middle panels). Both isoforms were present in prostate adenocarcinoma, but canonical MED19 expression was more prominent (histoscore 4 + 3 for canonical MED19 and 3 + 2 for alternative MED19) (Fig. 2A; bottom panels). The average histoscores for normal prostate (n = 27) and prostate adenocarcinoma (n = 18) samples stained with MED19 isoform-specific antibodies are shown in Supplementary Fig. S1. Our findings suggest that both isoforms are expressed at comparable protein levels during prostate development, and that canonical MED19 protein was expressed at a higher level than alternative MED19 in normal adult prostate and prostate adenocarcinoma.

We also analyzed endogenous canonical MED19 and alternative MED19 protein levels across prostate cell lines by performing western blots on total protein lysates using MED19 isoform-specific antibodies. We observed robust expression of endogenous canonical MED19 protein in all examined prostate cell lines (Fig. 2B). Additionally, we detected the presence of endogenous alternative MED19 protein, albeit at lower levels compared to canonical MED19. The expression of alternative MED19 protein was evident in LNCaP (an androgen-dependent line)^20^, LNCaP-abl^21^ and LNCaP-95^22^ (androgen-independent lines), and VCaP (an androgen-dependent line originating from a metastatic lumbar vertebral body lesion)^23^. VCaP cells displayed the highest expression of the alternative MED19 protein among the tested cell lines, whereas immortalized but not transformed normal adult prostate RWPE1 cells expressed the lowest. This suggests that multiple prostate cancer cell lines express both MED19 protein isoforms, mirroring the expression pattern observed in prostate tissue. Such a pattern implies that each isoform may play distinct roles within the context of the Mediator complex to modulate gene expression and phenotypic response.

It is important to note that the smaller MED19 isoform detected in the alternative MED19 blot does not result from canonical MED19 degradation. This conclusion is supported by the lack of cross-reactivity between the alternative MED19 antibody and canonical MED19 protein (Fig. 1B) and the inclusion of protease inhibitors in the cell lysis buffer^24^.

### Canonical MED19 promotes androgen independence

**Figure 3:**
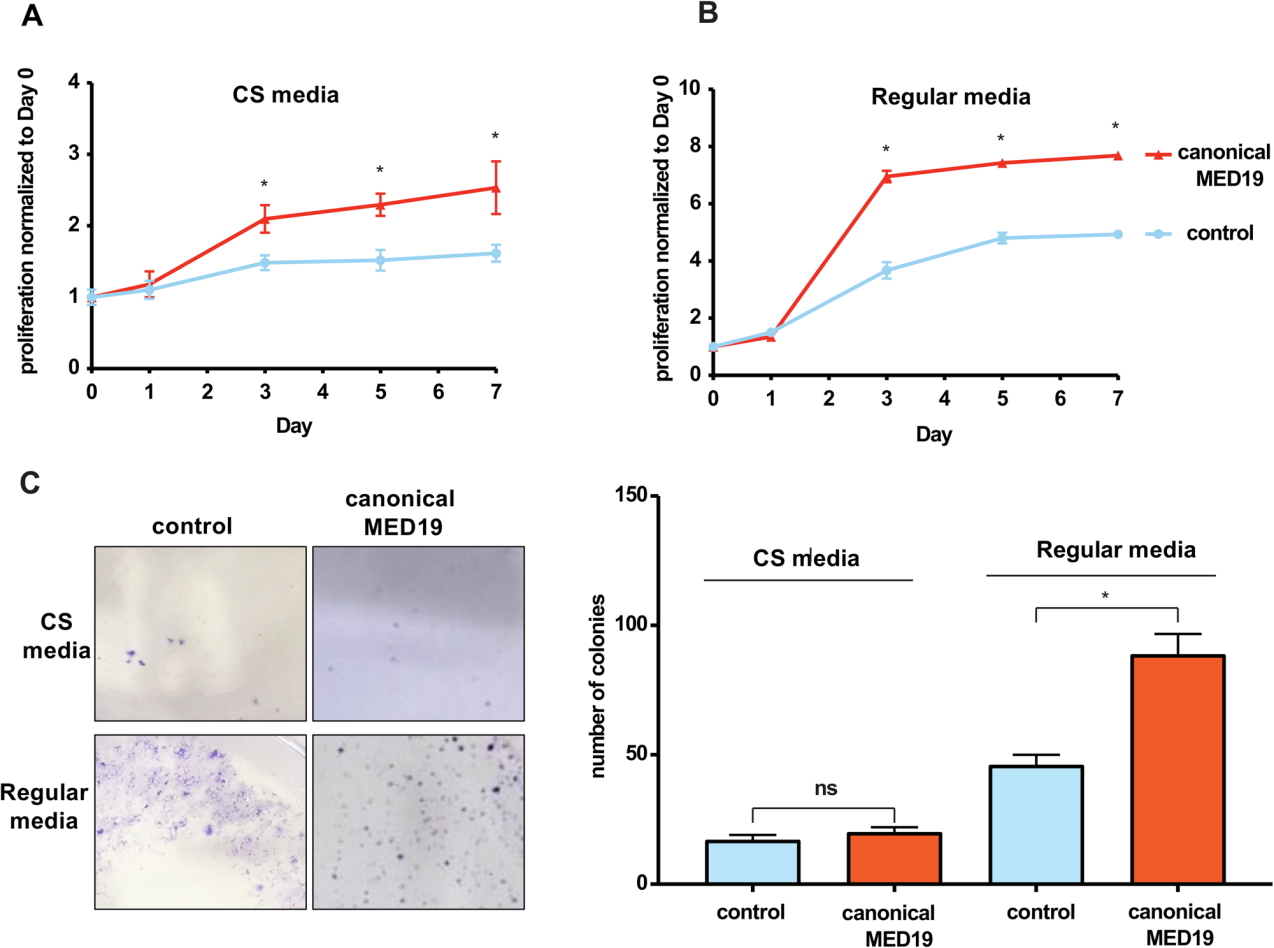
Canonical MED19 enhances proliferation but not colony formation under androgen deprivation. (**A**) LNCaP cells stably overexpressing canonical MED19 (red) or control empty vector (blue) were cultured in media depleted of androgens by using FBS charcoal-stripped of steroids (CS media) or (**B**) in media containing androgens by using standard FBS (regular media). Proliferation was assessed over 7 days using CyQUANT™ assay and is represented as the fold change in relative fluorescent units (RFU), normalized to Day 0 (n = 3; *p < 0.05). (**C**) Colony formation was analyzed by culturing canonical MED19 LNCaP, and control LNCaP cells at low density for 11 days in media depleted of androgens (CS media) or containing androgens (regular media), then fixed and stained with crystal violet. The number of colonies per field was quantified (n = 5; *p < 0.05), *ns* not significant). Pictures of the colony formation assay are shown, with LNCaP control cells previously published in Weber et al.^19^, and reproduced under the terms of the Creative Commons Attribution License.

We investigated whether the upregulation of canonical MED19 provided a proliferative advantage to prostate cancer cells. We used lentiviral transduction to increase the expression of canonical MED19 in androgen-dependent LNCaP cells. Additionally, we applied this method to an AKT-transformed mouse stem cell (MSC) line. This was done to demonstrate that the impact of canonical MED19 is not restricted to LNCaP cells, as we had earlier established with alternative MED19^19^. Our findings indicated that the expression of canonical MED19 was sufficient to promote proliferation in media depleted of androgens by using FBS charcoal-stripped of steroids (CS media) (Fig. 3A)^19^. Moreover, when cells were cultured in media containing androgens by using standard FBS (regular media), canonical MED19 overexpression also conferred a growth advantage (Fig. 3B). The AKT-MSC also grew markedly faster upon canonical MED19 overexpression compared to its control counterpart (Supplementary Fig. S2). This corroborates the growth advantage of canonical MED19 found in LNCaP cells and demonstrates that the overexpression of canonical MED19 is sufficient to promote androgen-independent and androgen-dependent prostate cancer cell proliferation.

We next assessed the survival and replicative potential of canonical MED19 LNCaP cells using a colony-formation assay^25^. This assay measures the ability of a single cell to proliferate and form a colony in vitro and is used to evaluate cell autonomous growth and metastatic potential of oncogenes^25^. We have previously demonstrated that alternative MED19 LNCaP cells exhibited robust colony formation compared to control LNCaP cells when seeded at a low density in androgen-deprived conditions^19^. However, there was a significant difference in colony-forming ability in cells overexpressing canonical MED19. Specifically, canonical MED19 LNCaP cells did not form colonies in androgen-depleted media, although colonies were capable of forming in regular media (Fig. 3C). These results imply that, in contrast to alternative MED19-expressing cells, canonical MED19-expressing cells are unable to support cell-autonomous growth under low androgen conditions.

We next investigated the dependence of canonical MED19 LNCaP cells on the AR for their androgen-independent proliferation by assessing their response to enzalutamide, an AR antagonist that reduces AR transcriptional activity^26^. As with alternative MED19 LNCaP cells^19^, enzalutamide reduced the proliferation of canonical MED19 LNCaP cells under androgen deprivation with an IC_50_ in the low micromolar range (Supplementary Fig. S3). This IC_50_ value is higher than reported values for LNCaP cells^26^, a discrepancy potentially due to the specific cell pools chosen affecting enzalutamide response. Consistent with this is our finding that the control LNCaP cells with an empty vector and selected with puromycin also show a similar IC_50_ value for enzalutamide to that of canonical MED19 LNCaP (this study) and alternative LNCaP cells (Weber et al*.*^19^). Additionally, we found that AR expression was comparable in canonical MED19 LNCaP cells compared to control LNCaP cells under androgen deprivation (Supplementary Fig. S3). Overexpression of canonical MED19 did not induce AR variant (AR-V7) expression (Supplementary Fig. S3). We also found that AR expression was equivalent between canonical MED19 cells and alternative MED19 LNCaP cells (Supplementary Fig. S3). Moreover, we found that the AR target gene PSA was upregulated in canonical MED19 LNCaP cells compared to control cells (Supplementary Fig. S3). Taken together, these findings suggest that the growth advantage provided by canonical MED19 relies on AR transcriptional activity and imply that canonical MED19 promotes androgen independence through AR.

**Figure 4:**
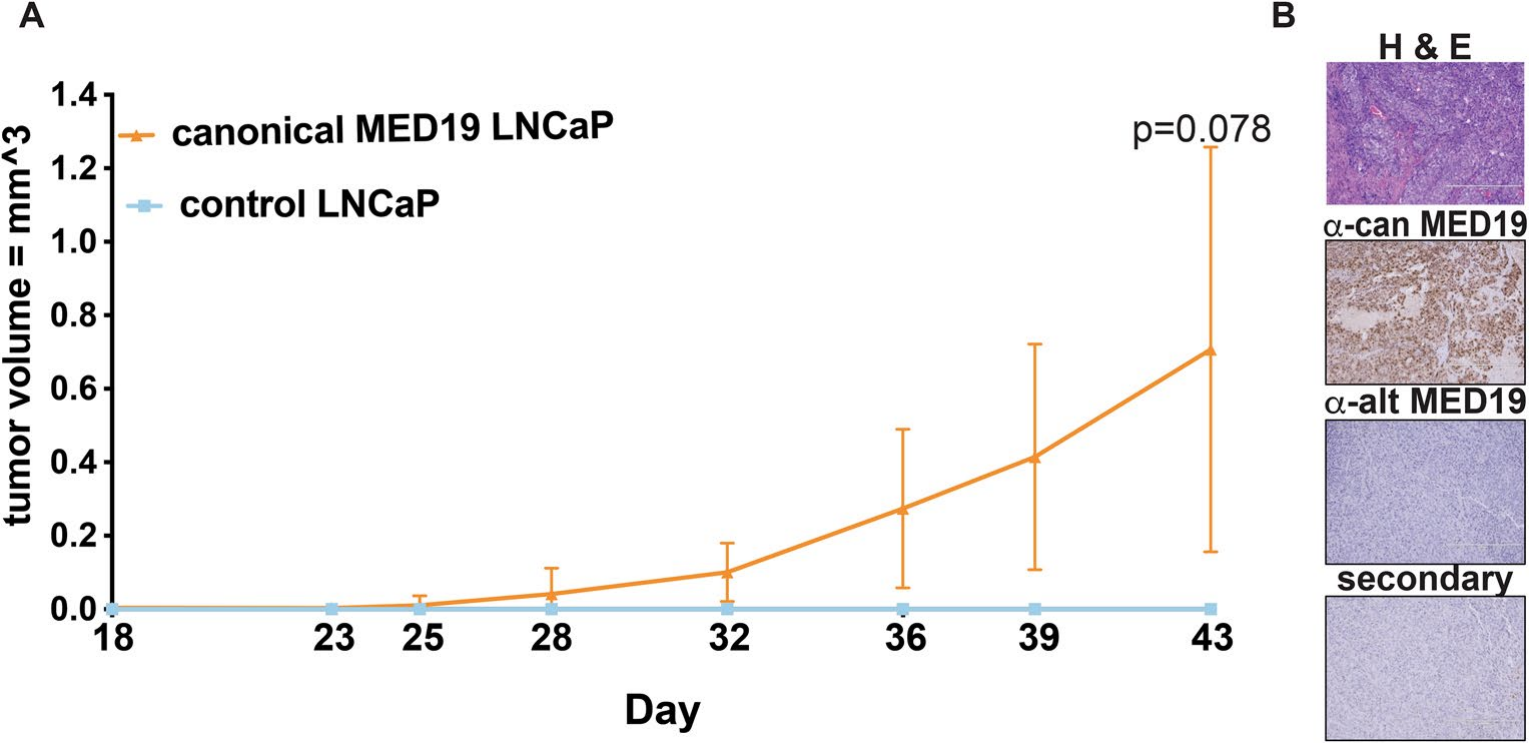
Canonical MED19 promotes tumor growth in castrated mice. (**A**) Castrated Nu/J mice were implanted with control LNCaP (blue) or canonical MED19 LNCaP cells (orange), and tumor size was measured on the indicated days (n = 4), p = 0.078. Area under the curve (AUC): canonical MED19 LNCaP = 2.5 ± 1.4; control LNCaP = 0.0. (**B**) Histology of canonical MED19 LNCaP tumors from castrated mice. H&E staining (top panel) of canonical MED19 LNCaP tumors revealed extensive necrotic areas (light pink). IHC staining of canonical MED19 LNCaP tumors using the indicated MED19 antibodies (middle panels). Secondary antibody alone control is shown in the bottom panel.

To investigate whether the proliferative capacity of canonical MED19 cells in vitro under androgen deprivation translates in vivo to increased tumor growth in castrate conditions, we conducted xenograft experiments. We implanted castrated Nu/J mice with either control LNCaP cells or canonical MED19 LNCaP cells subcutaneously. Canonical MED19 LNCaP cells formed sizable tumors, whereas the control LNCaP cells, due to their reliance on androgens for growth, did not form tumors in the castrated mice (Fig. 4). This observation was also replicated in an allograft model using AKT-MSC overexpressing canonical MED19 compared to its control counterpart (Supplementary Fig. S4). Therefore, the growth advantage that canonical MED19 provided under androgen-deprived conditions in vitro was also observed in vivo.

We conducted a histological examination of the canonical MED19 LNCaP tumors. Hematoxylin and eosin (H&E) staining displayed significant necrosis (light pink staining) (Fig. 4B), a sign of fast-growing tumors experiencing hypoxia and nutrient deprivation, leading to necrotic cell death^27^. We also performed immunohistochemistry (IHC) to examine MED19 isoform expression in vivo during tumor development. We observed a high level of canonical MED19 expression in the canonical MED19 LNCaP tumors, with minimal evidence of alternative MED19 (Fig. 4B). This implies that canonical MED19 expression is maintained within the tumors. Consequently, canonical MED19 provides a growth benefit to tumors and contributes to castration resistance.

### Canonical MED19 and alternative MED19 regulate unique sets of genes under androgen deprivation and with androgen treatment

Canonical and alternative MED19^19^ promote growth under low androgen conditions via AR transcriptional activity. Previous RNA-seq studies showed alternative MED19 alters gene expression under low androgen, upregulating the AR target gene monoamine oxidase A (MAOA), which promotes growth and metastasis^19,28^. We hypothesize that canonical MED19 similarly impacts gene expression during androgen deprivation, albeit with a unique transcriptome compared to cells expressing alternative MED19.

**Figure 5:**
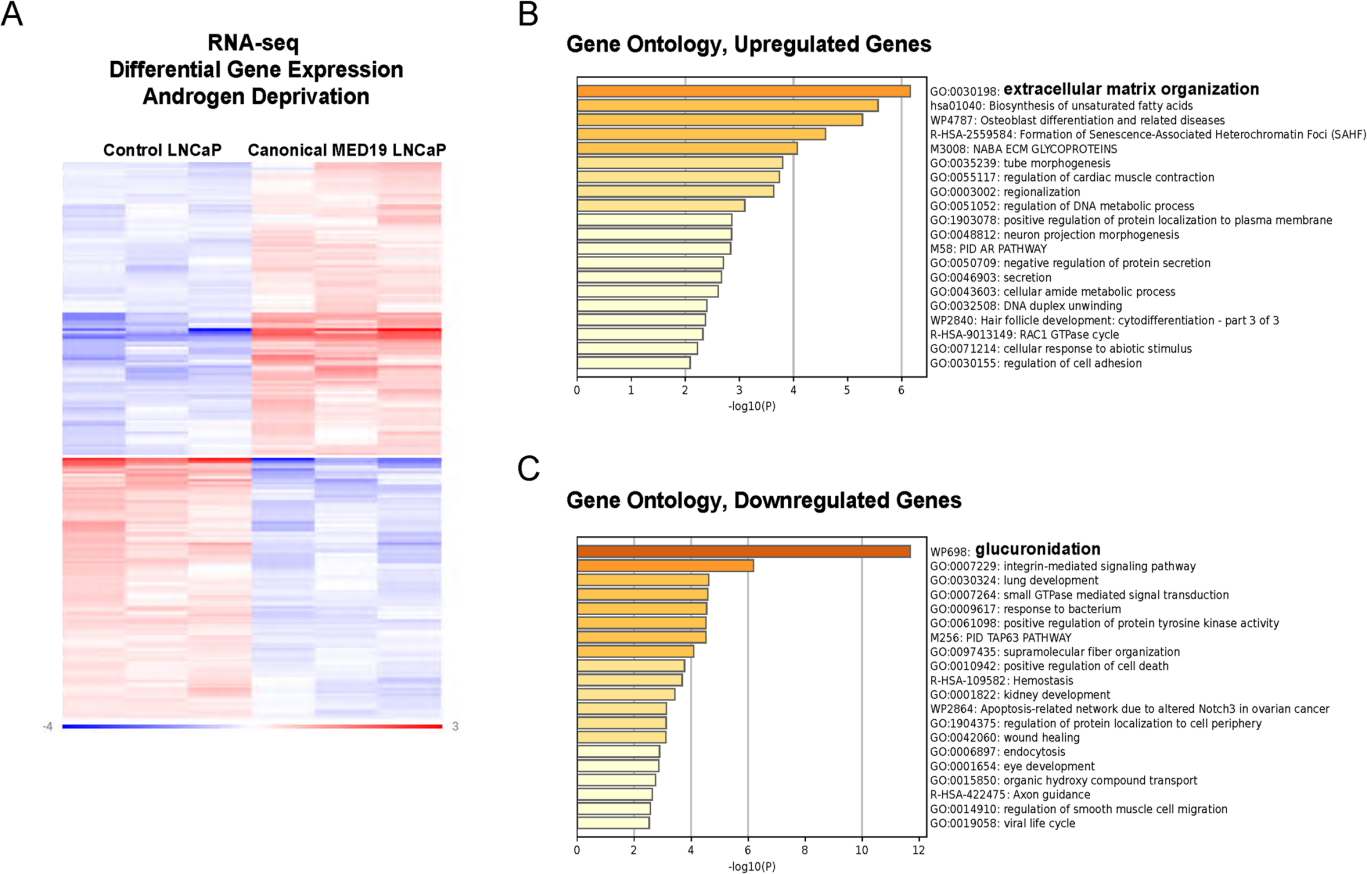
Overexpression of canonical MED19 in LNCaP cells leads to decreased expression of genes associated with glucuronidation under low androgen conditions. (**A**) Heatmap illustrating differentially expressed genes in canonical MED19 LNCaP cells compared to control LNCaP cells grown under androgen deprivation (209 genes: fold change ≥ 1.25, p ≤ 0.05). Clustering of the differentially expressed genes for the Heatmap was done by Rosalind using the PAM (Partitioning Around Medoids) method using the fpc R library https://cran.r-project.org/web/packages/fpc/index.html. (**B**) Metascape analysis of pathways involving upregulated and (**C**) downregulated genes in canonical MED19 LNCaP cells relative to control LNCaP cells.

To test this, we conducted RNA-seq from canonical MED19 LNCaP cells and control LNCaP cells. We determined the differentially expressed genes between control LNCaP and canonical MED19 LNCaP cells under androgen deprivation and compared those to the differentially expressed genes we previously identified from RNA-seq in alternative MED19 LNCaP cells versus control LNCaP cells under the same conditions^19^. Under androgen deprivation, the overexpression of canonical MED19 led to a significant and specific alteration in gene expression compared to control cells. This resulted in 209 genes being affected (110 upregulated and 99 downregulated; fold change ≥ 1.25 and p ≤ 0.05) (Fig. 5A). Notably, this is distinct from the differentially expressed genes when alternative MED19 is overexpressed, with only 22 genes overlapping (Supplementary Table S1). Pathway analysis using Metascape^29^ revealed that upregulated genes in canonical MED19 LNCaP cells compared to control LNCaP cells under androgen deprivation were related to extracellular matrix (ECM) organization^30^ and fatty acid biosynthesis^31^, both of which are known to play a role in prostate cancer progression (Fig. 5B). Interestingly, glucuronidation is the primary gene ontology category linked to the downregulated genes when canonical MED19 is overexpressed (Fig. 5C). This encompasses the well-established AR target genes UDP-glucuronosyltransferase (UGT) enzymes UGT2B15 and UGT2B17: enzymes involved in androgen inactivation via glucuronidation, whose expression is suppressed by AR^32,33^. Other UGT enzymes, including UGT2B7, UGT2B10, UGT2B11, and UGT2B28, were also downregulated. These results suggest that canonical MED19 inhibits androgen glucuronidation and inactivation, leading to increased expression of AR-regulated genes and promoting androgen-independent growth. Supporting this notion, overexpression of canonical MED19 under androgen deprivation led to increased expression of AR target genes such as KLK2, KLK3 (PSA), and KLK4 (Supplementary Table S1). We utilized Metascape pathway analysis to examine the RNA-seq results of alternative MED19 LNCaP cells versus control LNCaP cells cultured under androgen deprivation from our previous study^19^. The analysis revealed that upregulated genes were primarily associated with axon guidance, indicating increased cell motility (Supplementary Fig. S5). The downregulated genes were linked to NCAM1 interactions, implying potential changes in cell adhesion and differentiation that could be associated with metastatic potential (Supplementary Fig. S5). We also detected heightened cell movement in alternative MED19 LNCaP cells compared to control cells under androgen deprivation in vitro (Supplementary Fig. S6). Enhanced cell proliferation is unlikely to account for the increased migration observed in alternative MED19 cells since canonical MED19 overexpressing LNCaP cells also proliferate faster but do not show enhanced migration. Consequently, the genes and pathways influenced by canonical MED19 overexpression in LNCaP cells under androgen deprivation differ from those governed by alternative MED19, providing complementary oncogenic properties to prostate cancer cells.

Upon androgen treatment, we noted similar results: specifically, upon R1881 treatment, there were 2690 upregulated and 2633 downregulated genes in canonical MED19 LNCaP cells, compared to 2327 upregulated and 2510 downregulated genes in control LNCaP cells (fold change ≥ 1.25 and p ≤ 0.05). This is typical of the number of genes modulated by AR in this cell type^34^. In canonical MED19-overexpressing cells with R1881 treatment, there were 317 genes that were differentially expressed; 158 were upregulated and 159 were downregulated in comparison to control cells (Supplementary Fig. S7). These genes were distinct from those when alternative MED19 was overexpressed, with only 41 genes shared. When androgens were present, the principal gene ontology classes linked to canonical MED19 were the cell cycle (Supplementary Fig. S8), while for alternative MED19 (Supplementary Fig. S8), they were axon guidance. This suggests that canonical MED19 augments androgen-stimulated cell cycle progression, which aligns with its higher proliferative potential in complete media containing androgens (Fig. 3B).

### MED19 mRNA isoforms display varied expression across cancer types, with a higher expression of alternative MED19 observed in metastatic prostate cancer

**Figure 6:**
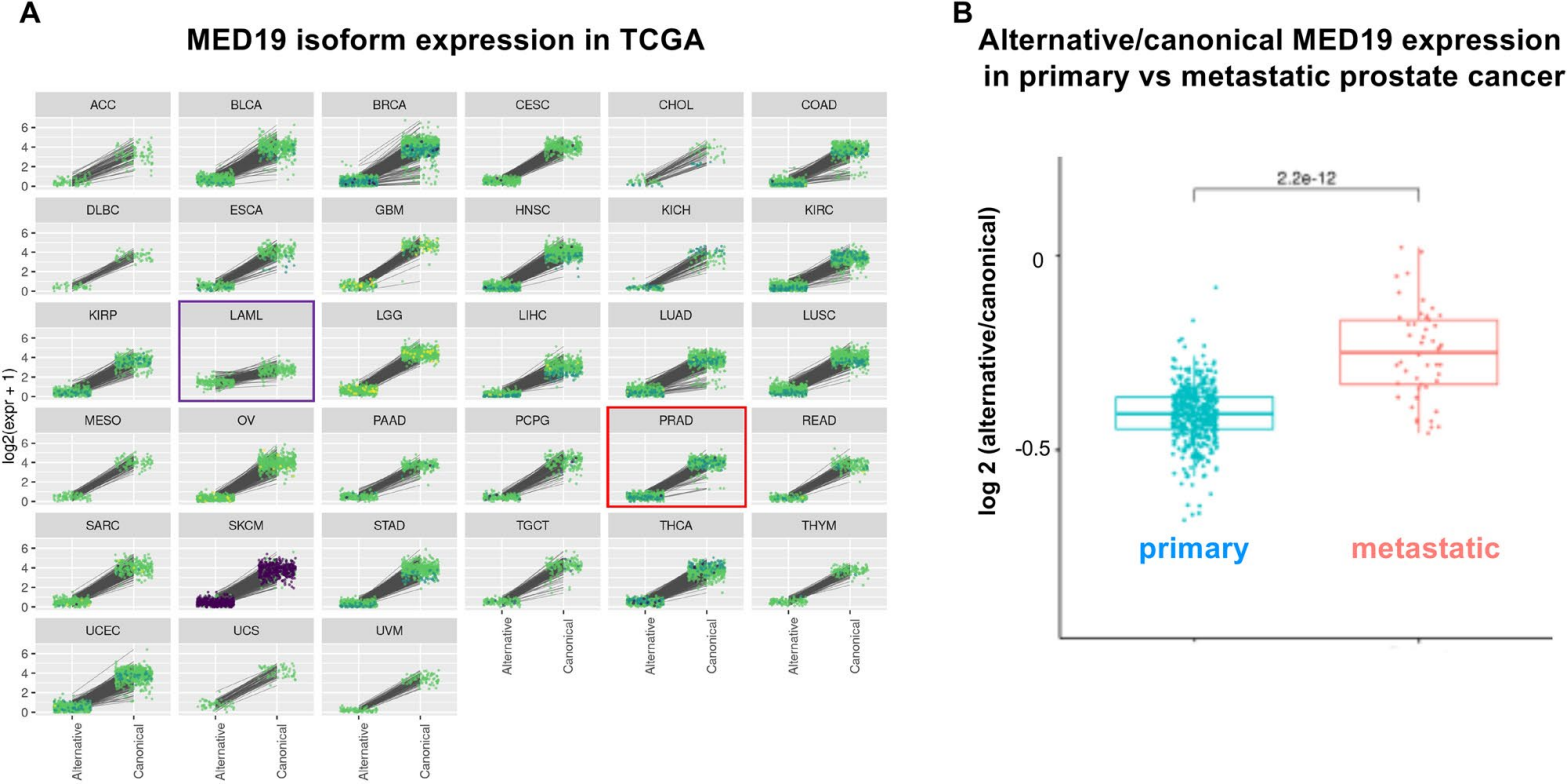
Canonical and alternative MED19 mRNA expression in primary and metastatic prostate cancer. (**A**) The expression of canonical and alternative MED19 isoforms across various cancer types. RNAseq data from TCGA was analyzed for the expression of canonical MED19 and alternative MED19 isoforms. Data are shown as log2 change of alternative/canonical MED19 expression. The red boxed area is prostate cancer (PRAD), and the purple boxed area is AML (LAML). (**B**) Comparison of alternative versus canonical isoform mRNA expression of MED19 in primary (TCGA) and metastatic prostate cancer from Beltran et al*.*^40^ was analyzed and presented as a box plot of log2 fold change. Primary samples: n = 499; metastatic samples: n = 49. The p-value is indicated.

MED19 has been demonstrated to impact cancer cell characteristics, including growth and mobility, across numerous cancers, including prostate cancer^3,35–38^. Previous studies have utilized siRNAs or antibodies that do not distinguish between MED19 isoforms. Consequently, we assessed the mRNA levels of MED19 variants using TCGA data and expressed the findings as a log2 ratio of alternative to canonical MED19 expression^39^. Across all cancer types, including prostate cancer (Fig. 6A; PRAD-red box), canonical MED19 is the more highly expressed isoform. While alternative MED19 is present in most cancers, it is typically expressed at lower levels compared to canonical MED19. A higher expression of alternative MED19 was detected in AML (Fig. 6A; LAML-purple box), but this was not associated with survival changes in AML.

We also evaluated the expression levels of MED19 isoforms in metastatic prostate cancer^40^. Given the unique impact of alternative MED19 on colony formation and cell migration, and its role in modifying genes associated with cell motility, adhesion, and differentiation^19^, we hypothesized an increased expression of alternative MED19 relative to canonical MED19 in metastatic samples. Indeed, we found that the ratio of alternative MED19 mRNA to canonical MED19 was greater in metastatic samples compared to non-metastatic samples (Fig. 6B). Additionally, the expression of both alternative and canonical MED19 protein are observed in a liver metastasis with an increased expression of alternative MED19 compared to canonical MED19 (Supplementary Fig. S9). Taken together, our findings suggest that MED19 isoforms are co-expressed in prostate cancer and other types of cancer. However, the ratio of canonical MED19 to alternative MED19 mRNA expression is higher in early-stage primary cancers, including prostate, whereas the ratio of alternative MED19 versus canonical MED19 is higher in later-stage prostate cancer metastasis. This is consistent with alternative MED19 overexpression facilitating cell-migration and cell-autonomous growth, both hallmarks of metastasis.

## Discussion

Here we demonstrate that MED19 generates two protein isoforms, each influencing gene expression differently and fostering prostate cancer cell growth and tumorigenesis in conditions of low androgen. The most straightforward explanation for how MED19 impacts gene expression is that each isoform can be incorporated into the Mediator complex to regulate distinct sets of genes. We propose that each MED19 isoform restructures the Mediator complex by influencing its interactions with other subunits in the middle module, resulting in new associations with specific transcription factors, thereby altering the expression of gene targets^16^. This idea is supported by our discovery that AR and ELK1 control alternative MED19-dependent gene expression in LNCaP cells^19^, whereas AR, CDX2, and FOXA1 are associated with canonical MED19-dependent gene expression under low androgen.

The regulatory pathway controlling MED19 expression remains unclear. Our analysis of the human MED19 locus from the ENCODE database reveals the presence of several transcriptional activators at the promoter region, including FOXA1, RELA, ETS, SRF, and members of the AP1 family, in addition to transcriptional repressors like REST^41^. These findings suggest that decreased repression by the loss of REST or increased activation of RELA (NF-kappa B) or AP1 by inflammatory signals and ETS and SRF via mitogenic signals could increase MED19 expression. This is supported by data from The Signaling Pathways Project^42^, which demonstrates a threefold increase in MED19 expression in the livers of male mice exposed to the inflammatory agent lipopolysaccharide (LPS) and a ninefold increase in human primary microvascular endothelial cells infected with MERS-CoV. Additionally, MED19, in conjunction with MED26, hinders REST recruitment to repressor elements, resulting in the de-repression of REST-target genes^43^. Further studies are required to understand the regulation of MED19 expression in prostate cancer.

**Figure 7:**
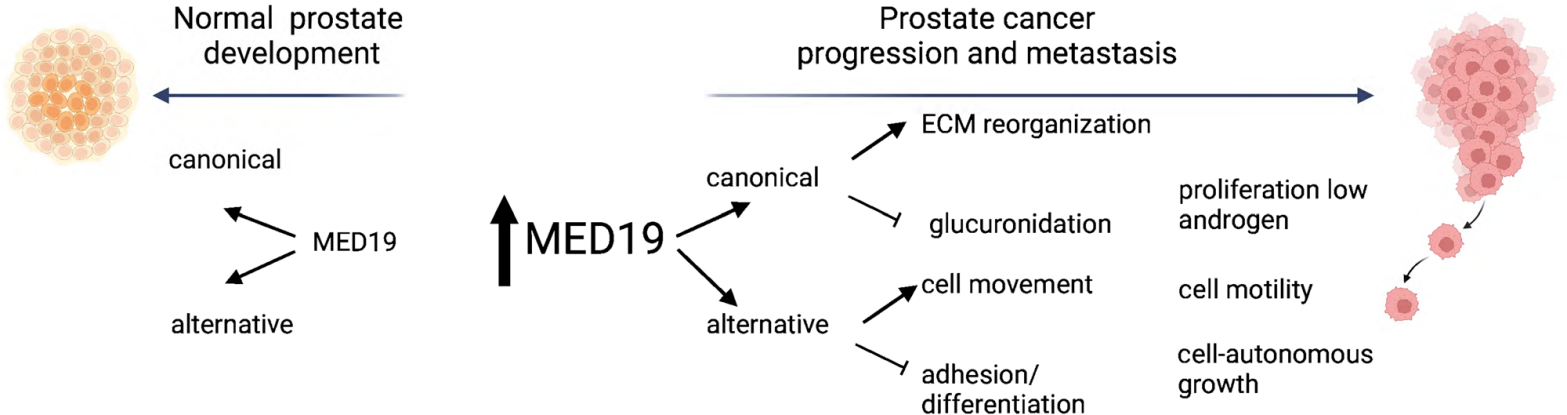
Distinct mechanisms of canonical and alternative MED19 dysregulation contribute to androgen independence and oncogenesis. This model illustrates the shift in MED19 isoform expression from balanced levels in prostate development to abnormal expression during prostate cancer progression and metastasis. Expression of MED19 isoforms is tightly regulated during the development of prostate epithelial cells. However, when this control is disrupted, an overexpression of MED19 isoforms occurs, leading to enhanced AR activity and prostate cell growth, even under low androgen conditions.

We propose that during normal prostate development a critical equilibrium is maintained between MED19 isoforms, a balance essential for prostate proliferation and differentiation. Disruptions causing increased MED19 expression could elevate AR activity, thereby increasing prostate cell growth, especially under conditions of androgen deprivation (Fig. 7). Significantly, when there is a rise in alternative MED19 expression, cells acquire enhanced motility and cell autonomous growth capabilities, characteristics vital for metastatic propagation^44^.

Furthermore, when canonical MED19 expression increases, it inhibits enzymes involved in glucuronidation, which are integral to deactivating androgens and thereby limiting androgen-independent cell growth. A pivotal study from the Sharifi lab illustrated that castration-resistant C42 cells, in comparison to castration-sensitive LNCaP cells, have a glucuronidation deficiency, enabling them to proliferate in low androgen conditions^45^. Consistent with this observation, we find an increase in canonical MED19 protein expression in C42 cells compared to LNCaP cells (Supplementary Fig. S10), suggesting its role in proliferation under low androgen due to reduced glucuronidation enzyme expression.

Our research establishes, for the first time, the presence of MED19 isoforms at the protein level and illuminates their singular roles in the progression of prostate cancer. These findings underscore the potential of MED19 as a promising therapeutic target across all stages of the disease^3^.

## Methods

### Cell culture

LNCaP (CRL-1740), PC3 (CRL-1435), DU-145 (HTB-81), VCaP (CRL-2876), 22Rv1 (CRL-2505), C4-2 (CRL-3314) and E006AA-hT (CRL-3277), cell lines were purchased from the ATCC. LNCaP-abl and LNCaP-95 cell lines were generous gifts from Z. Culig and R. Reiter, respectively. The mouse prostate stem cell (MSC) line expressing activated AKT is described in Ref.^46^ and was generously provided by Dr. Elaine Wilson, NYU School of Medicine. Cells were maintained as follows: LNCaP, 22Rv1 and C4-2 (RPMI 1640, 10% FBS), PC3 (Ham’s F-12 Nutrient Mixture, 10% FBS), LNCaP-95 and LNCaP-abl (RPMI 1640, phenol red-free, 10% charcoal dextran stripped FBS), DU-145, VCaP, E006AA (DMEM, 10% FBS). The MSC cell line expressing activated AKT was cultured as described^47^. Cells are routinely screened for mycoplasma.

For assays under androgen deprivation, cells were cultured in androgen-depleted RPMI: phenol red- and l-glutamine-free RPMI-1640 supplemented with 10% charcoal-stripped FBS (c-FBS, Hyclone) and 1% l-glutamine (Cellgro, Mediatech, Inc.). Cells were cultured on poly-d-lysine-coated plates. R1881 (Perkin Elmer) was reconstituted in ethanol. Enzalutamide (MedKoo) was reconstituted in DMSO. Puromycin (Sigma Aldrich) was reconstituted in water.

### Generation of prostate cancer cells with stable overexpression of canonical MED19

Canonical MED19 (NM_001317078.3 cDNA was purchased from Origene Technologies in the plenti-myc-DDK-P2A-puro expression vector. This results in MED19 proteins with N-terminal Myc and FLAG epitope tags. Lentiviral particles were produced in the 293T/17 cell line (ATCC CRL-11268). LNCaP and mouse prostate stem cell (MSC) lines were infected on two consecutive days with control vector only or canonical MED19 lentiviral particles and polybrene. Selection with puromycin (1 μg/mL) generated pooled clones. Canonical MED19 expression was verified by western blot. Alternative MED19-expressing LNCaP cells were generated as previously described^19^.

### Proliferation assay

Cells were plated in the appropriate media in quintuplicate (LNCaP: 3000 cells per well in complete media and 5000 cells per well in androgen-depleted media; MSC: 1000 cells per well) in poly-d-lysine-coated 96-well plates. Cell proliferation was determined using the Cyquant-NF Cell Proliferation Assay (ThermoFisher Scientific Cat# C35006) or PrestoBlue Cell Viability Assay (ThermoFisher Scientific Cat# A13261). Fluorescence was quantified with the SpectraMaxM5 Microplate Reader and SoftMaxPro software (Molecular Devices) and normalized to readings at Day 0 (the day after plating).

### Colony formation assay

LNCaP cells expressing MED19 isoforms were plated in duplicate at 5000 cells per well in complete media or 10,000 cells per well in androgen-depleted media on poly-d-lysine-coated 6-well plates for 10–14 days. Cells were fixed with 66% methanol/33% acetic acid solution and stained with 0.1% crystal violet.

### MED19 isoform-specific antibody production

Peptides corresponding to each MED19 isoform were synthesized by AnaSpec Inc. (San Jose, CA): MED19 canonical ^222^KNRHSPDHPGMGS^234^; MED19 alternative ^188^VPPGKPS^194^. A cysteine residue was added to the N termini of the peptides to facilitate chemical cross-linking. Before immunization, the peptide was coupled to keyhole limpet hemocyanin (Labcorp Research Products, Denver, PA). Three rabbits were immunized with each peptide, and the serum from each was tested by western blot. Sera with the greatest immunoreactivity were affinity-purified from a column prepared with the immunizing peptide (Labcorp Research Products).

### Xenograft/allograft studies

For in vivo experiments, control LNCaP and canonical MED19 LNCaP cells or mouse prostate stem cells expressing the empty vector or canonical MED19 isoform (5 × 10^6^) were mixed with an equal volume of Matrigel and injected subcutaneously into the flank region of Nu/J castrated male mice (Jackson Laboratories). Tumor volume was measured twice weekly. All animal studies were performed at the NYU School of Medicine. The animal research was approved by the NYU School of Medicine Institutional Animal Care and Use Committee (IACUC). All experiments comply with the ARRIVE guidelines.

### Immunohistochemistry

Immunohistochemistry was performed using the affinity-purified MED19 isoform-specific antibodies. A prostate specimen from a human fetus at 23 weeks of gestational age was obtained following surgical termination for reasons unrelated to this investigation. Approval for collecting samples was received by the New York University Grossman School of Medicine Institutional Review Board. Benign adult prostate tissues and prostate adenocarcinoma specimens were obtained from a prostate cancer tissue microarray (TMA) (US Biolab). Metastatic prostate cancer samples from the 15 Case Metastasis from Rapid Autopsy TMA were obtained from the Prostate Cancer Biorepository Network (PCBN). Immunohistochemistry (IHC) was performed on paraffin‐embedded tissue sections that were dewaxed in xylene, rehydrated, and washed in phosphate‐buffered saline (pH 7.4). Antigen retrieval was performed by heating sections in a microwave oven (900 watts) for 20 min in 10 mM citrate buffer, followed by treatment with 3% H_2_O_2_ and blocked with 20% normal goat serum. Sections were incubated for 24 h at 4 °C with antibodies against canonical MED19 (1:100) or alternative MED19 (1:100), washed, and followed by a 1-h incubation with a biotinylated rabbit secondary (1:1000; Vector Labs). An avidin‐biotin complex was formed and developed using diaminobenzidine chromagen, followed by a counterstain with hematoxylin.

### Protein extraction and western blot analysis

Cells were lysed in RIPA buffer supplemented with a protease inhibitor cocktail (Cell Signaling Technology; cat # 5781). Protein lysates were subjected to SDS/PAGE and immunoblotted with antibodies against the MED19 isoforms or MYC tag (Cell Signaling Technology; cat # 2276S). Tubulin (Covance; cat # MMS-489P) was used as a loading control. Protein bands were visualized using a Clarity Western ECL Substrate (BioRad; cat #1705060), and images were acquired on an iBright CL1000 (ThermoFisher Scientific) using the “Auto-Exposure” option in the iBright Imaging System software. This feature utilizes software algorithms to analyze all signals present within a selected frame automatically. Imaging parameters are as follows: Zoom level = 1.2×; Focus level = 246; Resolution = 5 × 5; Exposure mode = normal; Exposure times vary depending on the antibody and amount of immunogen: 0.3–10 s.

### RNA sequencing

RNA sequencing has been previously described in Weber et al.^19^. Total RNA was extracted using RNeasy (Qiagen; cat #74104) according to the manufacturer’s instructions. Libraries were prepared with ribodepletion using Illumina TruSeq stranded total RNA with RiboZero Gold library preparation kit. Sequencing was performed using the Illumina HiSeq2500 Sequencing system (HiSeq 4000 Paired-End 50 or PE75 Cycle Lane). Data were analyzed by ROSALIND® (https://rosalind.bio/), with a HyperScale architecture developed by ROSALIND, Inc. (San Diego, CA). Reads were trimmed using cutadapt. Quality scores were assessed using FastQC. Reads were aligned to the *Homo sapiens* genome build hg19 using STAR. Individual sample reads were quantified using HTseq and normalized via Relative Log Expression (RLE) using DESeq2 R library. Read Distribution percentages, violin plots, identity heatmaps, and sample MDS plots were generated as part of the QC step using RSeQC. DEseq2 was also used to calculate fold changes and p-values and perform optional covariate correction. Clustering of genes for the final Heatmap of differentially expressed genes was done using the PAM (Partitioning Around Medoids) method using the fpc R library; https://cran.r-project.org/web/packages/fpc/index.html. Hypergeometric distribution was used to analyze the enrichment of pathways, gene ontology, domain structure, and other ontologies. The topGO R library was used to determine local similarities and dependencies between GO terms in order to perform Elim pruning correction. Several databases were referenced for enrichment analysis, including Interpro, NCBI, KEGG, MSigDB, REACTOME, WikiPathways. Enrichment was calculated relative to a set of background genes relevant to the experiment.

### MED19 isoform RNA expression analysis

MED19 RNA expression in TCGA (The Cancer Genome Atlas) and Beltran et al.^40^ tumor samples were downloaded from https://osf.io/gqrz9/files. This data set was part of a study benchmarking data analysis time using google cloud^39^. FASTQ sequencing files were processed with Kallisto (https://pachterlab.github.io/kallisto/) using Gencode v24 annotations to estimate transcript-level RNA expression. TPM (transcripts per million) values for all downstream analyses. Expression of transcript ENST00000431606.2 and ENST00000337672.6 were used to represent canonical and alternative isoforms for MED19, respectively.

### Statistical analyses

Statistical analyses were performed using GraphPad Prism software. Data are reported as mean ± SEM (technical replicates for each experiment described above). The number of experiments is described in the figure legends; unless otherwise noted, a two-tailed unpaired Student’s t-test was used when comparing two groups, with a p-value < 0.05 being considered significant and levels of significance denoted as *p < 0.05; **p < 0.01; and ***p < 0.001.

### Supplementary Information

Supplementary Figures.

Supplementary Table S1.

## Data Availability

Data generated or analyzed during this study will be available from the corresponding authors upon reasonable request. The RNA seq datasets of canonical MED19 LNCaP cells generated and analyzed during the current study are available in the NCBI/ GEO repository under GSE236441.

## References

[1] Harper TM, Taatjes DJ (2018). The complex structure and function of mediator. J. Biol. Chem..

[2] Lambert E, Puwakdandawa K, Tao YF, Robert F (2021). From structure to molecular condensates: Emerging mechanisms for Mediator function. FEBS J..

[3] Weber H, Garabedian MJ (2018). The mediator complex in genomic and non-genomic signaling in cancer. Steroids.

[4] Conaway RC, Conaway JW (2013). The mediator complex and transcription elongation. Biochim. Biophys. Acta.

[5] Chen Z (2022). Phosphorylated MED1 links transcription recycling and cancer growth. Nucleic Acids Res..

[6] Soutourina J (2018). Transcription regulation by the mediator complex. Nat. Rev. Mol. Cell Biol..

[7] Rengachari S, Schilbach S, Cramer P (2023). Mediator structure and function in transcription initiation. Biol. Chem..

[8] Imberg-Kazdan K (2013). A genome-wide RNA interference screen identifies new regulators of androgen receptor function in prostate cancer cells. Genome Res..

[9] Yu S (2017). Knockdown of mediator complex subunit 19 suppresses the growth and invasion of prostate cancer cells. PLoS ONE.

[10] Rengachari S, Schilbach S, Aibara S, Dienemann C, Cramer P (2021). Structure of the human mediator-RNA polymerase II pre-initiation complex. Nature.

[11] Zhao H (2021). Structure of mammalian mediator complex reveals Tail module architecture and interaction with a conserved core. Nat. Commun..

[12] Zhang H (2021). Mediator structure and conformation change. Mol. Cell.

[13] Chen X (2021). Structures of the human mediator and mediator-bound preinitiation complex. Science.

[14] Schilbach S (2017). Structures of transcription pre-initiation complex with TFIIH and mediator. Nature.

[15] Robinson PJ (2016). Structure of a complete mediator-RNA polymerase II pre-initiation complex. Cell.

[16] El Khattabi L (2019). A pliable mediator acts as a functional rather than an architectural bridge between promoters and enhancers. Cell.

[17] Rienzo M (2012). Distinct alternative splicing patterns of mediator subunit genes during endothelial progenitor cell differentiation. Biochimie.

[18] Cui X (2011). Suppression of MED19 expression by shRNA induces inhibition of cell proliferation and tumorigenesis in human prostate cancer cells. BMB Rep..

[19] Weber H, Ruoff R, Garabedian MJ (2021). MED19 alters AR occupancy and gene expression in prostate cancer cells, driving MAOA expression and growth under low androgen. PLoS Genet..

[20] Horoszewicz JS (1983). LNCaP model of human prostatic carcinoma. Cancer Res..

[21] Culig Z (1999). Switch from antagonist to agonist of the androgen receptor bicalutamide is associated with prostate tumour progression in a new model system. Br. J. Cancer.

[22] Leung JK, Tam T, Wang J, Sadar MD (2021). Isolation and characterization of castration-resistant prostate cancer LNCaP95 clones. Hum. Cell.

[23] Korenchuk S (2001). VCaP, a cell-based model system of human prostate cancer. In Vivo.

[24] Briggs EM (2018). Long interspersed nuclear element-1 expression and retrotransposition in prostate cancer cells. Mob. DNA.

[25] Franken NA, Rodermond HM, Stap J, Haveman J, van Bree C (2006). Clonogenic assay of cells in vitro. Nat. Protoc..

[26] Tran C (2009). Development of a second-generation antiandrogen for treatment of advanced prostate cancer. Science.

[27] Lee SY (2018). Regulation of tumor progression by programmed necrosis. Oxid. Med. Cell Longev..

[28] Wei J (2021). Bidirectional cross-talk between MAOA and AR promotes hormone-dependent and castration-resistant prostate cancer. Cancer Res..

[29] Zhou Y (2019). Metascape provides a biologist-oriented resource for the analysis of systems-level datasets. Nat. Commun..

[30] Stewart DA, Cooper CR, Sikes RA (2004). Changes in extracellular matrix (ECM) and ECM-associated proteins in the metastatic progression of prostate cancer. Reprod. Biol. Endocrinol..

[31] Sena LA, Denmeade SR (2021). Fatty acid synthesis in prostate cancer: Vulnerability or epiphenomenon?. Cancer Res..

[32] Grosse L (2013). Androgen glucuronidation: An unexpected target for androgen deprivation therapy, with prognosis and diagnostic implications. Cancer Res..

[33] Bao BY (2008). Androgen receptor mediates the expression of UDP-glucuronosyltransferase 2 B15 and B17 genes. Prostate.

[34] Labbe DP, Brown M (2018). Transcriptional regulation in prostate cancer. Cold Spring Harb. Perspect. Med..

[35] Ding XF, Huang GM, Shi Y, Li JA, Fang XD (2012). Med19 promotes gastric cancer progression and cellular growth. Gene.

[36] Sun M (2011). MED19 promotes proliferation and tumorigenesis of lung cancer. Mol. Cell Biochem..

[37] Zhang H (2012). Expression of Med19 in bladder cancer tissues and its role on bladder cancer cell growth. Urol. Oncol..

[38] Li LH, He J, Hua D, Guo ZJ, Gao Q (2011). Lentivirus-mediated inhibition of Med19 suppresses growth of breast cancer cells in vitro. Cancer Chemother. Pharmacol..

[39] Tatlow PJ, Piccolo SR (2016). A cloud-based workflow to quantify transcript-expression levels in public cancer compendia. Sci. Rep..

[40] Beltran H (2016). Divergent clonal evolution of castration-resistant neuroendocrine prostate cancer. Nat. Med..

[41] Svensson C (2014). REST mediates androgen receptor actions on gene repression and predicts early recurrence of prostate cancer. Nucleic Acids Res..

[42] Ochsner SA (2019). The signaling pathways project, an integrated ‘omics knowledgebase for mammalian cellular signaling pathways. Sci. Data.

[43] Ding N (2009). MED19 and MED26 are synergistic functional targets of the RE1 silencing transcription factor in epigenetic silencing of neuronal gene expression. J. Biol. Chem..

[44] Gerstberger S, Jiang Q, Ganesh K (2023). Metastasis. Cell.

[45] Zhu Z (2018). Loss of dihydrotestosterone-inactivation activity promotes prostate cancer castration resistance detectable by functional imaging. J. Biol. Chem..

[46] Xiong X (2018). KLF4, a gene regulating prostate stem cell homeostasis, is a barrier to malignant progression and predictor of good prognosis in prostate cancer. Cell Rep..

[47] Salm SN (2005). TGF-{beta} maintains dormancy of prostatic stem cells in the proximal region of ducts. J. Cell Biol..

