# Supplementary material for: MED19 encodes two unique protein isoforms that confer prostate cancer growth under low androgen through distinct gene expression programs
Source: Sci Rep. 2023 Oct 25;13:18227. doi: 10.1038/s41598-023-45199-9 (PMC10600210; doi:10.1038/s41598-023-45199-9)
Supplement: Supplementary file 1 — Supplementary Figures. [file 41598_2023_45199_MOESM1_ESM.pdf]

SUPPLEMENTARY INFORMATION FILE

Title: MED19 encodes two unique protein isoforms that confer prostate cancer growth under low androgen through distinct gene expression programs

Authors list: Rachel Ruoff, Hannah Weber, Ying Wang, Hongying Huang, Ellen Shapiro, David Fenyö and Michael J. Garabedian

Supplementary Figure 1

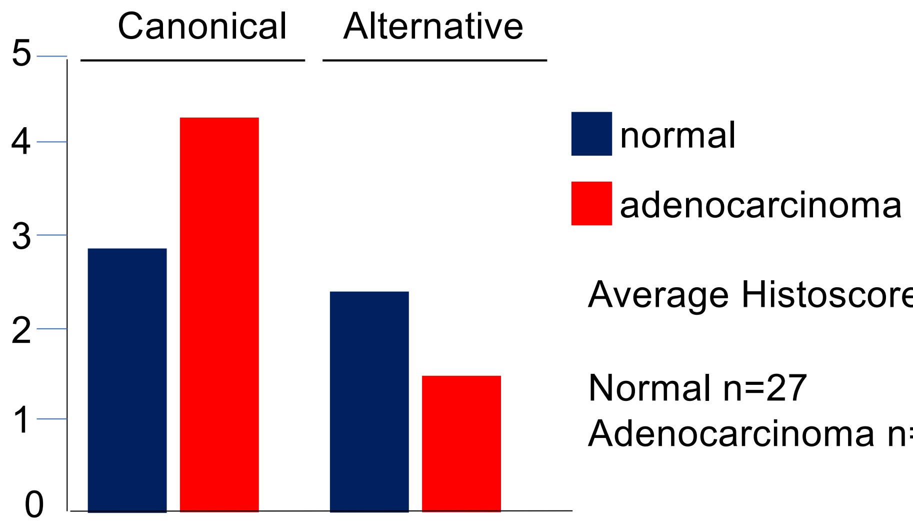

Average Histoscores

	Can	Alt
Normal n=27	3.0	2.4
Adenocarcinoma n=18	4.4	1.5

Figure S1. Average histoscores for the staining of MED19 isoforms in normal prostate and prostate adenocarcinoma. Shown are the average histoscores for the normal prostate (n=27) and prostate adenocarcinoma (n=18) from a tissue microarray (TMA) stained with either canonical MED19 or alternative MED19 antibodies. The score represents the mean value of the individual histoscores obtained from the samples.

Supplementary Figure 2

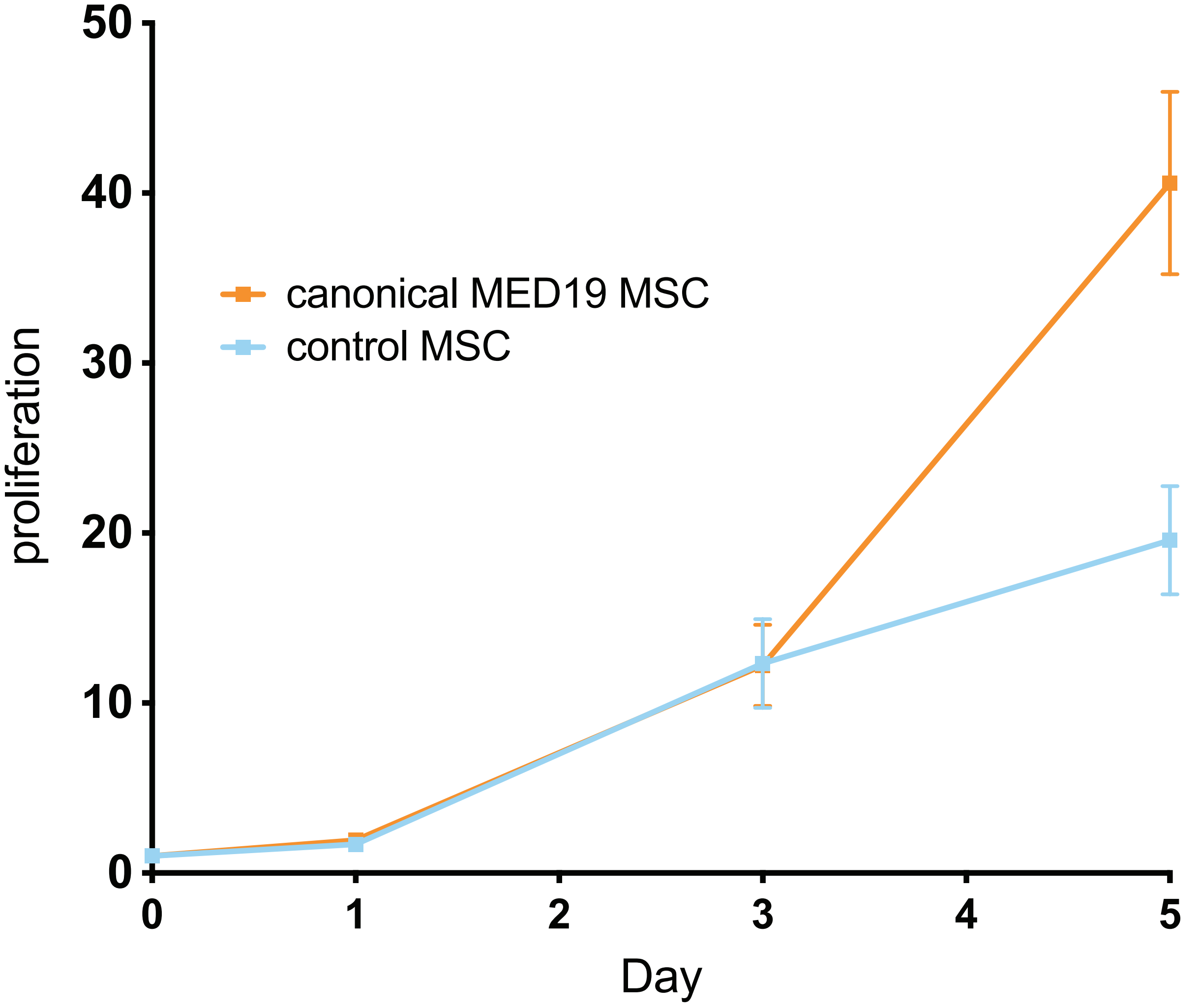

Figure S2. The proliferation of mouse prostate stem cells in vitro is enhanced by overexpression of canonical MED19
Mouse stem cells (MSC) that express a constitutively active myristoylated AKT and stably overexpressing canonical MED19 (canonical MED19 MSC, orange) or a control empty vector (control MSC, blue) were cultured in media containing 10% FBS and DHT. After 5 days, proliferation was measured using CyQUANT™ and is represented as a fold change in relative fluorescent units (RFU) normalized to Day 0.

Supplementary Figure 3

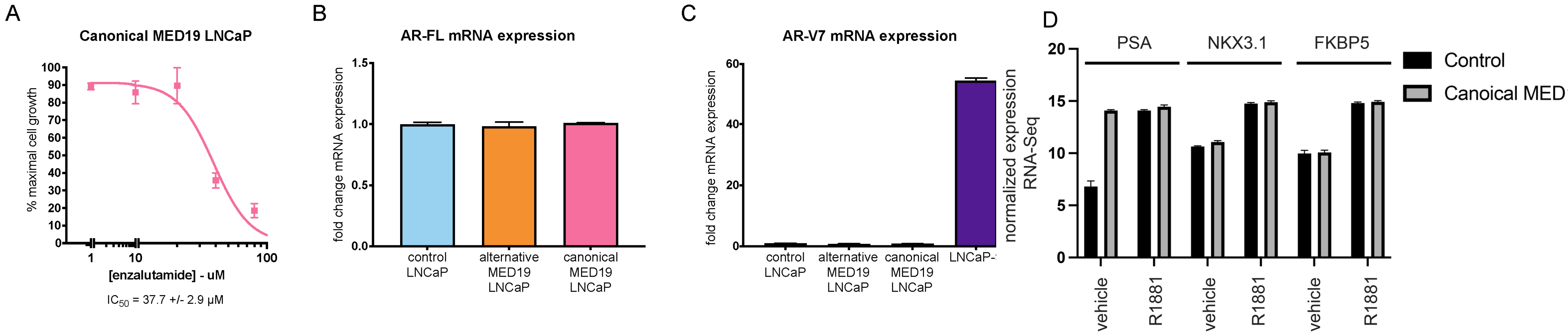

Figure S3. Androgen-independent growth occurs in canonical MED19 LNCaP cells without a concurrent rise in full-length AR expression or enhancement of AR-V7, and selective effects of canonical MED19 on AR target gene expression.
A) Cell proliferation of canonical MED19 LNCaP cells was measured at day 7 in androgen-depleted media with enzalutamide treatment ranging from 0-80 μM. The IC_{50} is shown (n=2; error bars represent the range of the mean). B-C) RNA was extracted from control LNCaP, canonical MED19 LNCaP, and alternative MED19 LNCaP cells cultured under androgen deprivation for 3 days. qPCR was used to measure the mRNA expression of full-length AR and AR-V7, with fold change normalized to RPL19. LNCaP-95 cells (purple) are a positive control for AR V7 expression. The experiments were performed in triplicate. D) Expression of AR target genes in control and canonical MED19 LNCaP cells from the RNA seq data from the vehicle and R1881 treated cells.

Supplementary Figure 4

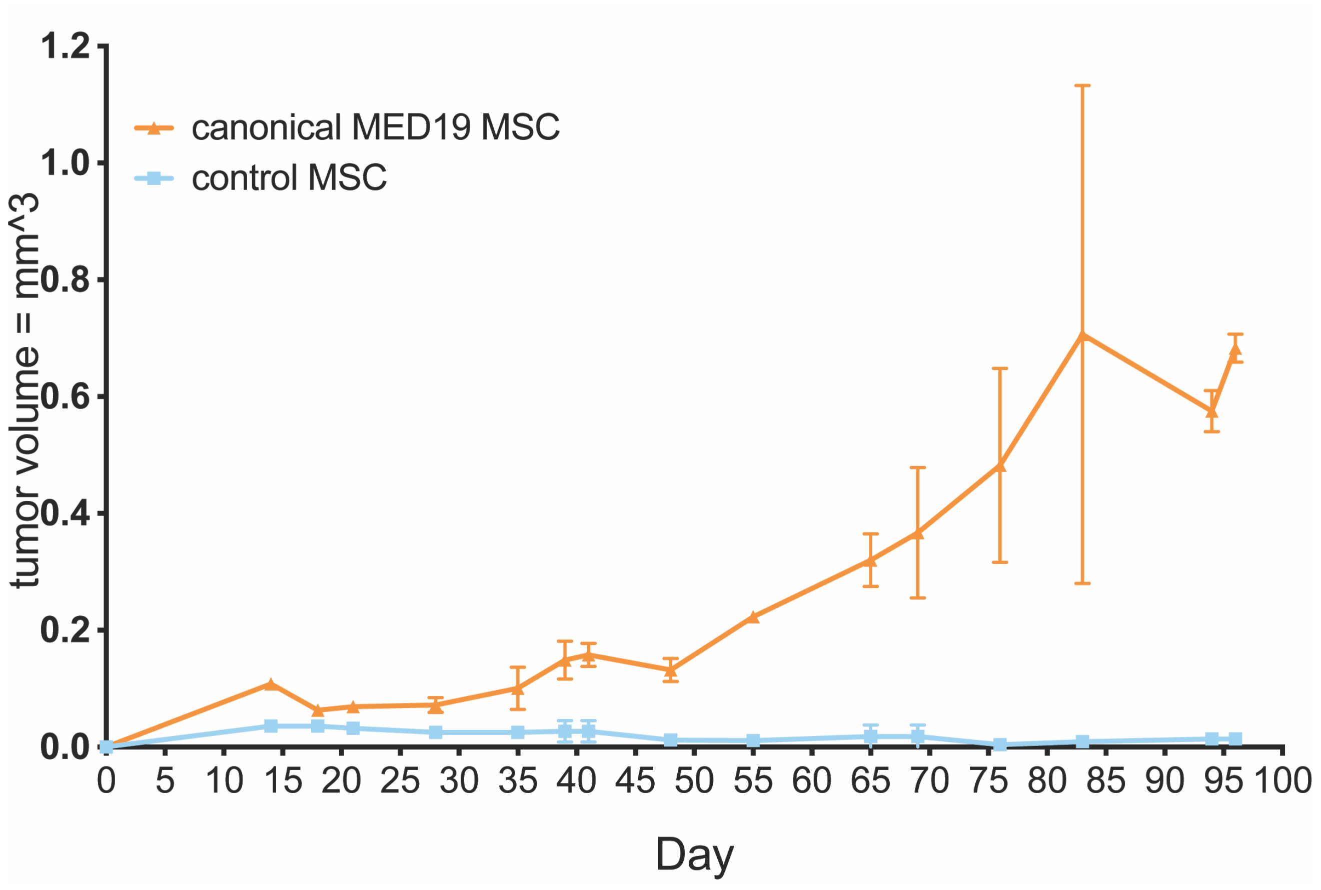

Figure S4. Canonical MED19 promotes tumor growth in AKT-transformed mouse stem cells (MSC) under conditions of low androgen
Castrated Nu/J mice were subcutaneously implanted with control MSC (blue) and canonical MED19 MSC (orange), and tumor size was measured at the indicated days (n=4).

Supplementary Figure 5

A **Alternative MED19 LNCaP, Androgen Deprivation**
Gene Ontology, Upregulated Genes

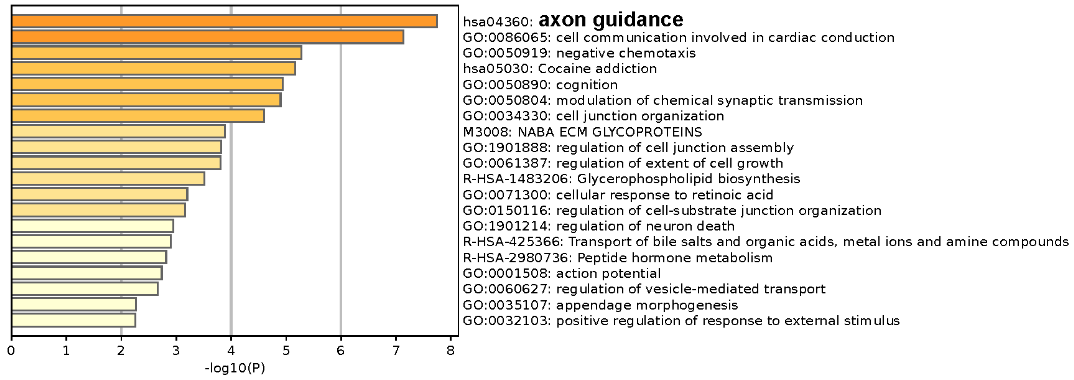

B **Alternative MED19 LNCaP, Androgen Deprivation**
Gene Ontology, Downregulated Genes

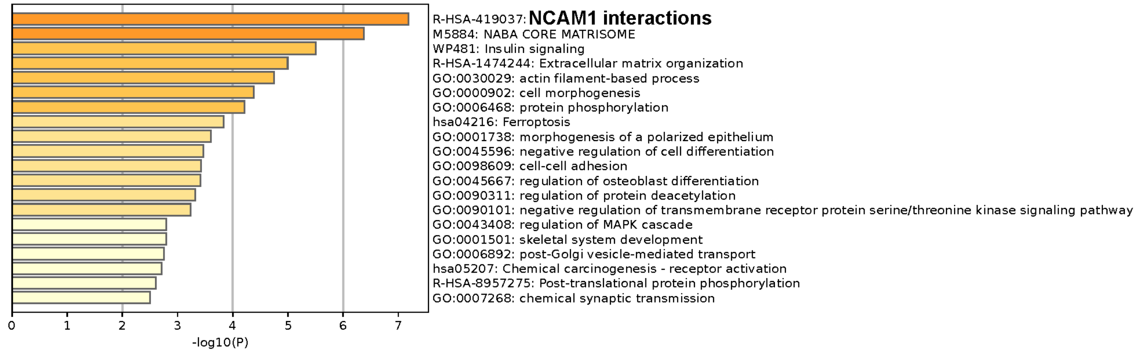

Figure S5. Pathway analysis of alternative MED19 LNCaP cells under low androgen
Metascape analysis of gene ontology (GO) pathways associated with genes A) upregulated and B) downregulated in alternative MED19 LNCaP cells relative to control LNCaP cells under androgen deprivation.

Supplementary Figure 6

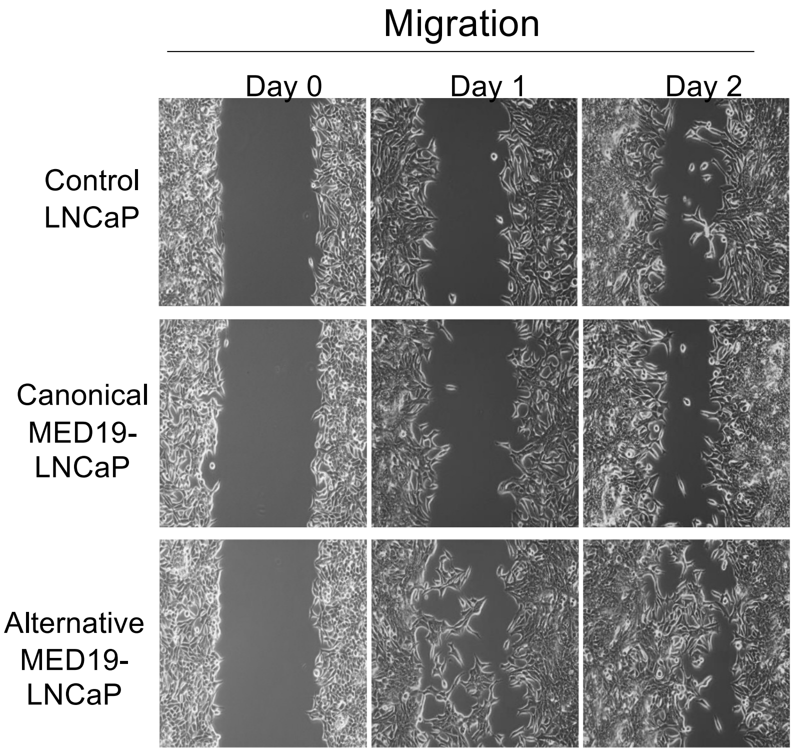

Figure S6. Alternative MED19 overexpression promotes migration in LNCaP cells. Control LNCaP, canonical MED19 LNCaP, and alternative MED19 LNCaP cells were evaluated for cell migration using a scratch assay under low androgen conditions. Micrographs of the cells are shown on days 0, 1, and 2. Shown is a representative experiment that was repeated twice.

Supplementary Figure 7

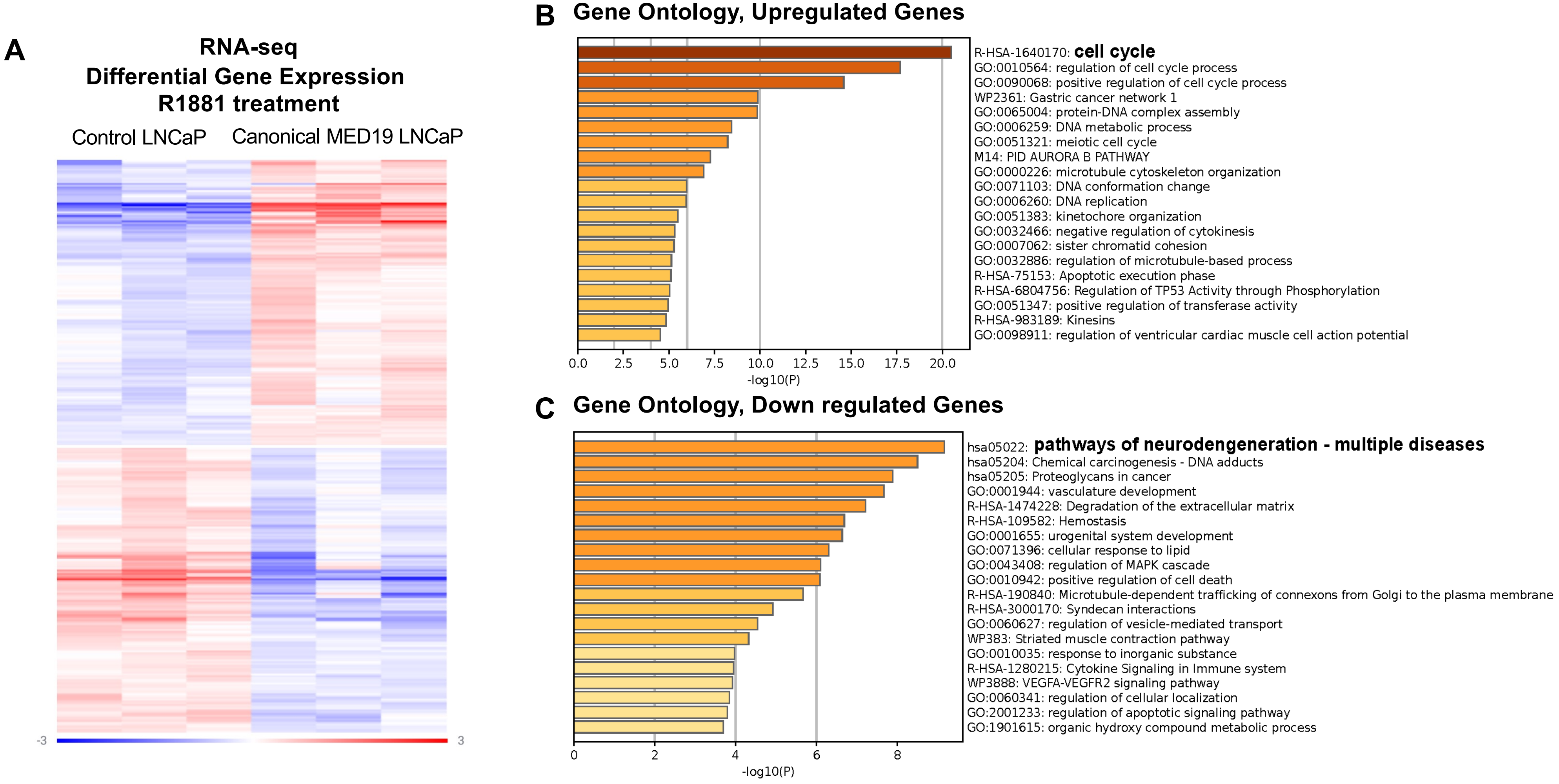

Figure S7. Canonical MED19 overexpression in LNCaP cells stimulates the expression of cell cycle-related genes upon androgen treatment. A) A heatmap illustrates the differential expression of 317 genes (fold change of ≥1.25 and p-value of ≤0.05) between control LNCaP cells and canonical MED19 LNCaP following R1881 treatment (10 nM, 16 h). Pathway analysis was performed on the B) upregulated and C) downregulated genes in canonical MED19 LNCaP cells versus control LNCaP cells using Metascape.

Supplementary Figure 8

A Alternative MED19 LNCaP, R1881 treatment
Gene Ontology, Upregulated Genes

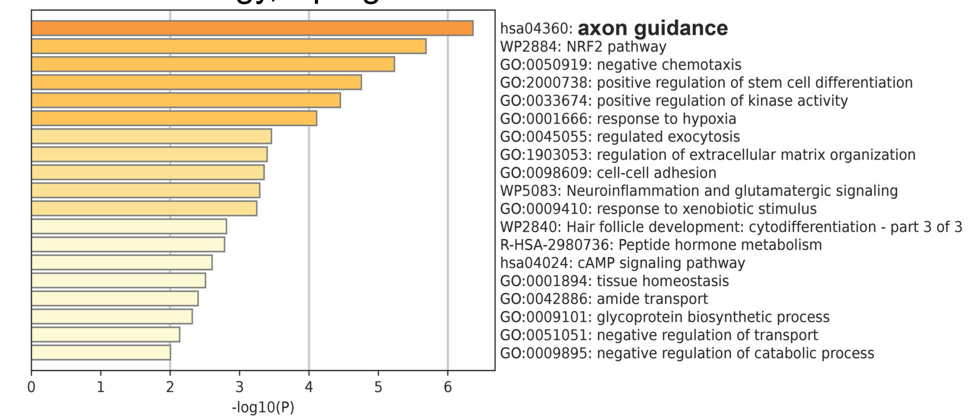

B Alternative MED19 LNCaP, R1881 treatment
Gene Ontology, Downregulated Genes

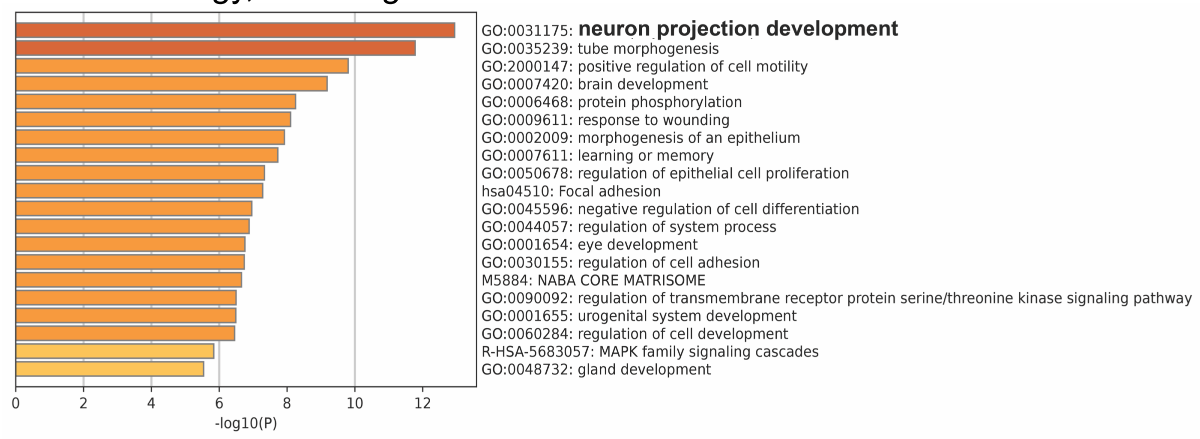

Figure S8. Pathway analysis of alternative MED19 LNCaP cells upon androgen treatment
Metascape analysis of gene ontology (GO) pathways associated with genes A) upregulated and B) downregulated in alternative MED19 LNCaP cells relative to control LNCaP cells following R1881 treatment (10 nM, 16 h).

Supplementary Figure 9

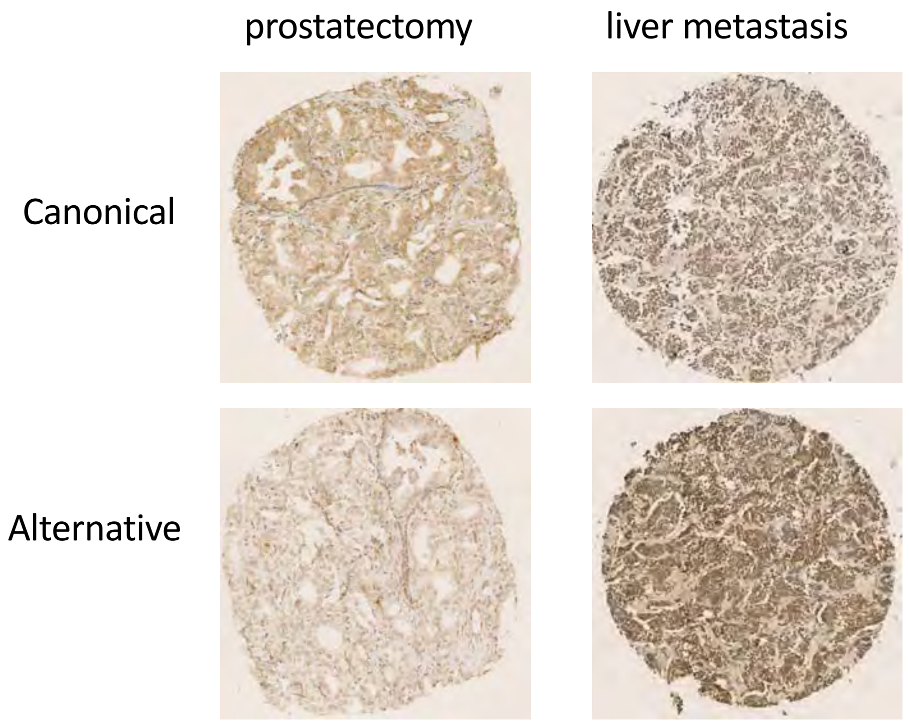

Figure S9. MED19 isoform protein abundance in primary and metastatic samples.
Representative images of canonical and alternative MED19 stained tumor tissue from prostatectomy and liver metastasis.

Supplementary Figure 10

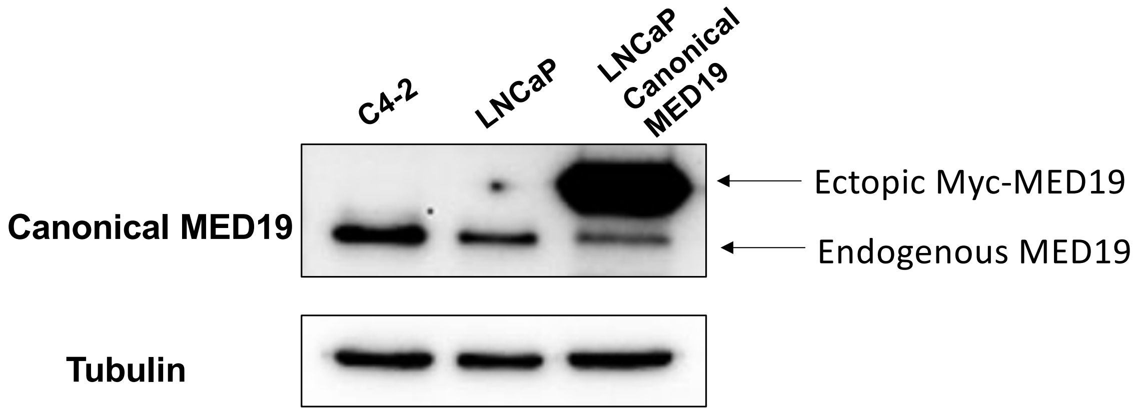

Figure S10. Canonical MED19 protein abundance is higher in C42 cells than in LNCaP cells. Western blot of C42, parental LNCaP, and LNCaP cells overexpressing canonical MED19 was blotted with canonical MED19 and tubulin as a loading control.

Supplementary Figure 11A

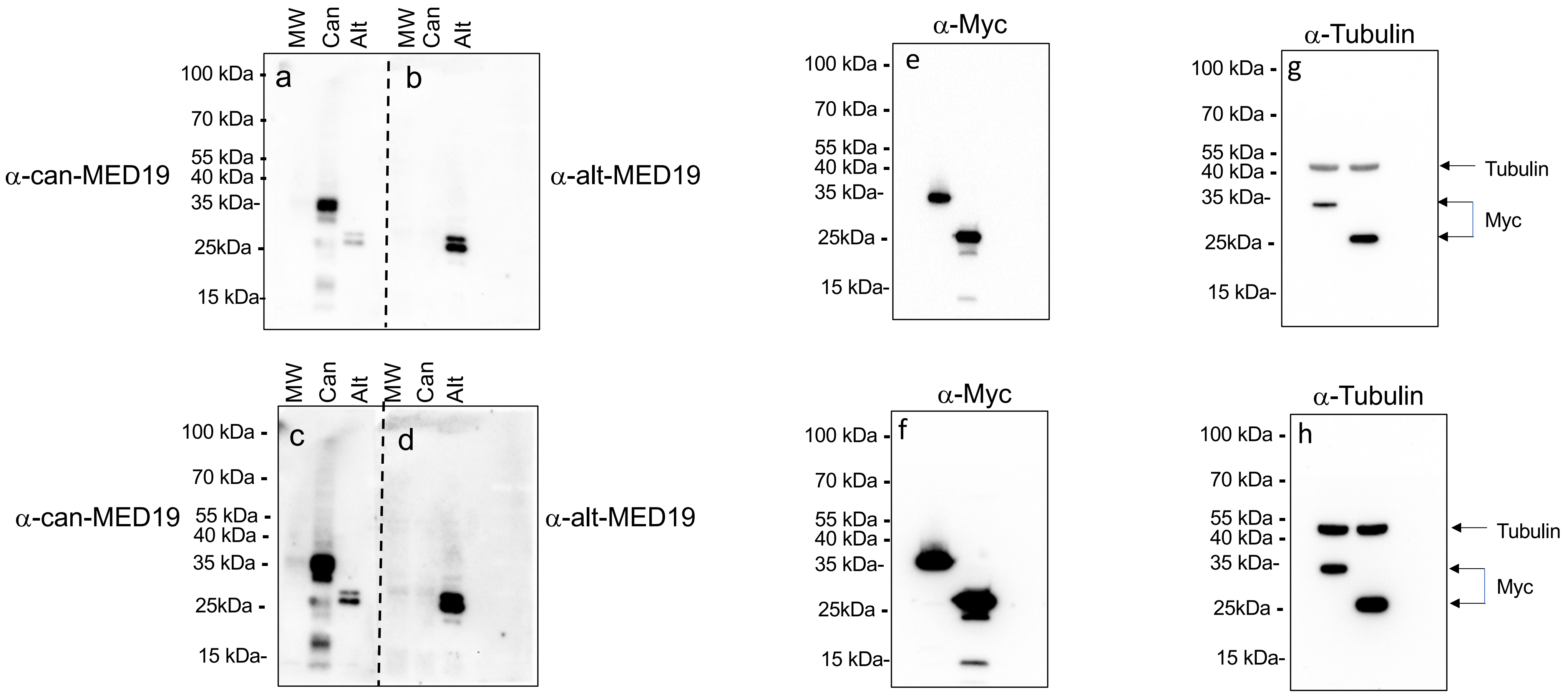

Figure S11: Uncropped western blots from Figure 1C: The same amount of whole cell lysates from LNCaP cells expressing canonical (Can) and alternative (Alt) MED19 were run in duplicate on the same gel, transferred to a membrane, cut in two (dashed line), and blotted separately with affinity purified antibody against canonical MED19 (panels a, c) or alternative MED19 (panels b, d). The individual blots were placed side by side and exposed for the same length of time (top panel=1.5 seconds; bottom panel 5.0 seconds). The membrane from panel b was then blotted with an antibody to the Myc tag common to both proteins (panels e, f). Tubulin (panel g, h) served as a loading control and was blotted after the Myc blot and shows the residual Myc-tagged MED19 protein expression. Shown are the raw images of two exposures using the Auto-Exposure option from the iBright Imaging System. MW=Molecular weight markers.

Supplementary Figure 11B

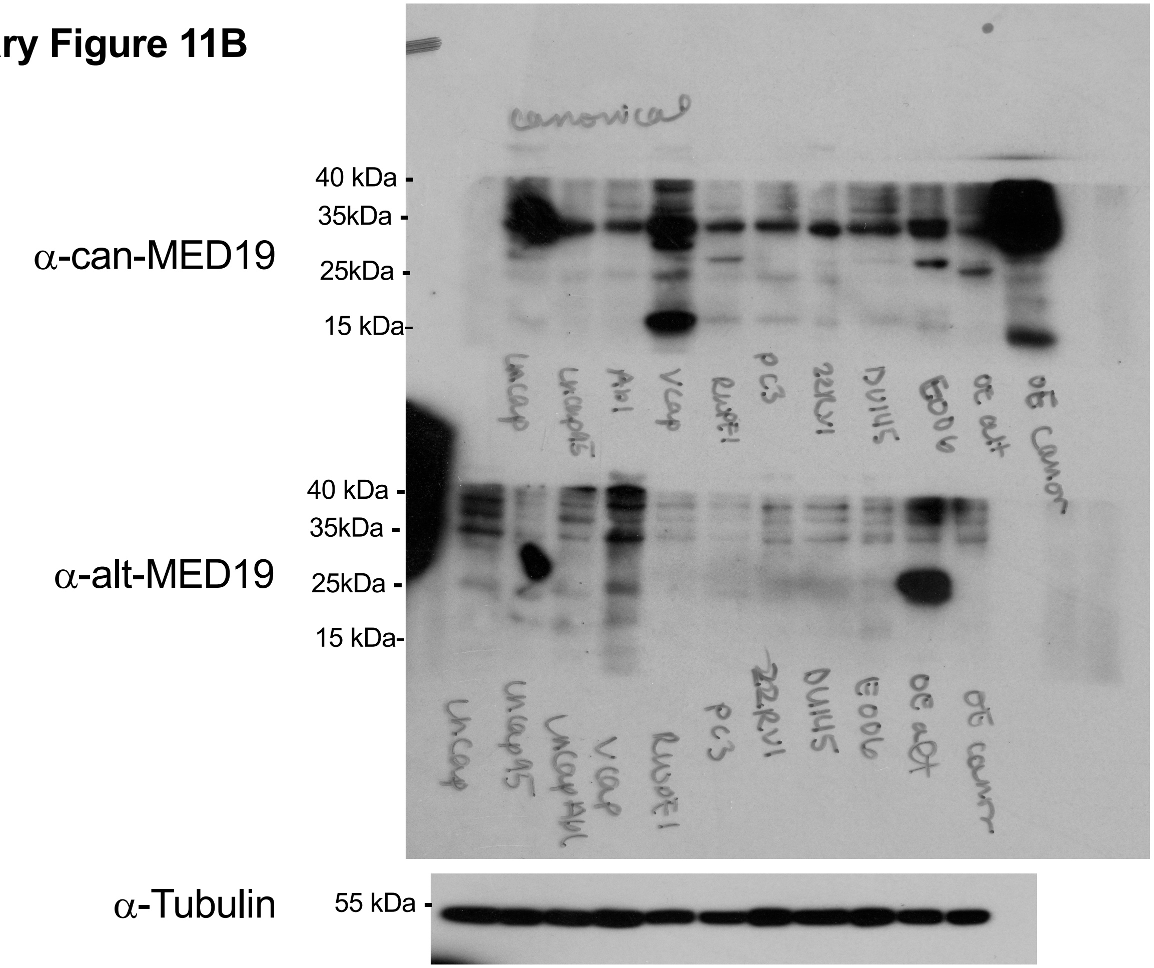

Figure S11B: Uncropped western blots from Figure 2B: Western blot of total protein lysates from the indicated prostate cancer cell lines (LNCaP, LNCaP-95, LNCaP-Abl, VCaP, RWPE1, PC3, 22Rv1, DU145, E006 (a kidney cancer cell line misidentified as a prostate cancer cell line), alternative MED19 LNCaP (OE alt) and canonical MED19 LNCaP; OE Can) using affinity-purified rabbit antibodies against canonical or alternative MED19. A horseradish peroxidase-conjugated goat anti-rabbit IgG secondary antibody was used. Blots were then washed three times for 10 min with TBS and 0.1% Triton X-100, twice with TBS, developed using enhanced chemiluminescence (ECL), and exposed to X-ray film. The top portion of the blot for alternative MED19 was cut prior to hybridization with the antibody against tubulin.

Supplementary Figure 11C

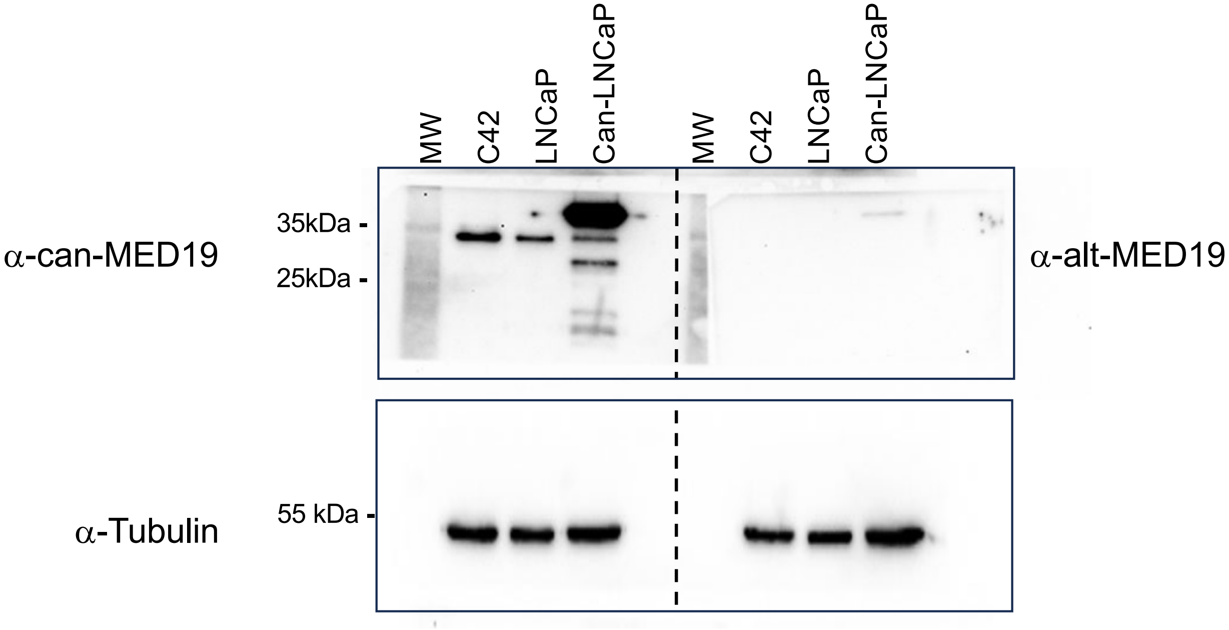

Figure S11C: Uncropped western blots from Figure S12: The same amount of whole cell lysates from C42, LNCaP, and LNCaP cells overexpressing canonical MED19 (Can-LNCaP) were run in duplicate on the same gel, transferred, cut in two (dotted line), and blotted separately with canonical MED19 (left side), alternative MED19 (right side), and tubulin as a loading control. The individual blots were placed side by side and exposed simultaneously. Chemiluminescent was captured via the iBright Imaging System using the Auto-Exposure option. Shown are the raw unprocessed images. The imaging parameters are as follows: Zoom level=1.2x; Focus level=246; Resolution=5x5; Exposure mode= normal; Exposure time: top panel 1.5 seconds; bottom panel = 0.3 seconds. MW=Molecular weight markers.
